# *Fungal* diversity from Fildes Peninsula (Antarctica) and their antibiosis bioactivity against two plant pathogens

**DOI:** 10.71150/jm.2411029

**Published:** 2025-04-14

**Authors:** Ji Seon Kim, Enzo Romero, Yoonhee Cho, Ramón Ahumada-Rudolph, Christian Núñez, Jonhatan Gómez-Espinoza, Ernesto Moya-Elizondo, Sigisfredo Garnica, Young Woon Lim, Jaime R. Cabrera-Pardo

**Affiliations:** 1 School of Biological Sciences and Institute of Biodiversity, Seoul National University, Seoul 08826, Republic of Korea; 2 Applied and Sustainable Chemistry Laboratory (LabQAS), Department of Chemistry, Universidad del Bío-Bío, Concepción 4051381, Chile; 3 Technical Professional High School Diego Portales, Department of Science, Linares, Chile; 4 Department of Plant Production, Faculty of Agronomy, Universidad de Concepción, Concepción 3812120, Chile; 5 Institute of Biochemistry and Microbiology, Faculty of Sciences, Universidad Austral de Chile, Teja Island, Valdivia 5090000, Chile; 6 College of Dental Medicine, Roseman University of Health Sciences, South Jordan, UT 84095, USA

**Keywords:** Antarctic fungi, antibiosis, *Botrytis cinerea*, *Fusarium culmorum*

## Abstract

Antarctic fungi can effectively adapt to extreme environments, which leads to the production of unique bioactive compounds. Studies on the discovery of fungi in the diverse environments of Antarctica and their potential applications are increasing, yet remain limited. In this study, fungi were isolated from various substrates on the Fildes Peninsula in Antarctica and screened for their antibiosis activity against two significant plant pathogenic fungi, *Botrytis cinerea* and *Fusarium culmorum*. Phylogenetic analysis using multiple genetic markers revealed that the isolated Antarctic fungal strains are diverse, some of which are novel, emphasizing the underexplored biodiversity of Antarctic fungi. These findings suggest that these fungi have potential for the development of new antifungal agents that can be applied in agriculture to manage fungal plant pathogens. Furthermore, the antibiosis activities of the isolated Antarctic fungi were evaluated using a dual-culture assay. The results indicated that several strains from the genera *Cyathicula*, *Penicillium*, and *Pseudeurotium* significantly inhibited pathogen growth, with *Penicillium pancosmium* showing the highest inhibitory activity against *Botrytis cinerea*. Similarly, *Aspergillus* and *Tolypocladium* strains exhibited strong antagonistic effects against *Fusarium culmorum*. This study enhances our understanding of Antarctic fungal diversity and highlights its potential for biotechnological applications.

## Introduction

Fungi are among the largest groups of organisms and thrive in diverse environments, where they occupy multiple ecological niches and play several roles, including saprotrophic, pathogenic, and symbiotic roles, making them essential ecosystem components (Kendrick, 2011; Naranjo-Ortiz and Gabaldón, 2019). Globally, their biomass accounts for approximately 12 gigatons of carbon (Bar‐On et al., 2018). Consequently, they exhibited a distinctive metabolic plasticity that enables rapid adaptation and survival through the biosynthesis of various natural products (Bhattarai et al., 2021; Gholami-Shabani et al., 2019). Fungi-derived natural products are pharmaceutically prolific and have been developed for several important biological applications, ranging from highly potent toxins to approved drugs (Aly et al., 2011; Rastegari et al., 2020; Schueffler and Anke, 2014; Vicente et al., 2003).

Antarctica represents one of the most extreme environments on Earth for the existence of life. This ecosystem exhibits high-stress conditions, including low temperatures, sporadic and limited nutrient availability, high aridity, and elevated ultraviolet radiation levels. Antarctic fungi must adapt to survive under these highly demanding conditions (Hassan et al., 2016). These adaptations result from modifications in gene expression and secondary metabolite biosynthesis, forming biologically relevant chemical spaces that allow them to survive efficiently in Antarctica (Varrella et al., 2021; Zucconi et al., 2020).

Studies on fungi in Antarctic ecosystems are limited. However, many studies on Antarctic fungi have explored the diversity and potential applications of culturable fungi from various Antarctic environments (González et al., 2020; Varrella et al., 2021). Despite these efforts, many studies on fungal diversity in Antarctica rely primarily on the internal transcribed spacer (ITS) region, a universal fungal genetic marker (Schoch et al., 2012), which often leads to inaccurate identification (Dupuis et al., 2012; Kiss, 2012). This difficulty in species identification limits our understanding of fungal biology and its potential applications.

Most studies on Antarctic fungi have focused primarily on the characteristics of secondary metabolites, including novel metabolite production and antibacterial properties (Ordóñez-Enireb et al., 2022; Shi et al., 2022; Vieira et al., 2018). However, their potential use, particularly in combating plant pathogens, remains undetermined. Recent findings regarding natural compounds that capable of inhibiting plant diseases have generated renewed interest (Kim and Hwang, 2007; Vinale et al., 2014; Wang et al., 2023). Therefore, Antarctic fungi may be promising candidates with hidden and remarkable capabilities.

*Botrytis cinerea* and *Fusarium culmorum* are representative plant pathogenic fungi that cause significant economic losses to agriculture. *Botrytis cinerea*, responsible for grey mold, is a highly destructive pathogen and is estimated to cause nearly $100 billion in annual agricultural losses (Dwivedi et al., 2024; Roca-Couso et al., 2021). The destructive nature of this fungus ranks second among scientifically and economically relevant pathogenic fungi (Dean et al., 2012). In Chile, *B. cinerea* affects grapes by reducing both yield and quality during ripening. Integrated management practices, including cultural, chemical, and biological methods, are crucial for controlling this pathogen in Chilean vineyards under temperate and humid conditions (Herrera-Défaz et al., 2023; Latorre et al., 2015).

*Fusarium culmorum*, on the other hand, affects cereals, such as wheat and barley, causing Fusarium head blight, which reduces grain yield and quality. The presence of this pathogen in Chile is significant, especially in humid areas where it can produce mycotoxins such as deoxynivalenol, posing additional food safety concerns (Scherm et al., 2013). Chemical strategies through fungicides are currently the most widely used methods for controlling infections. Both *B. cinerea* and *F. culmorum* have developed resistance to several conventional fungicides (Yin et al., 2023), causing substantial agricultural damage worldwide (Hahn, 2014). Therefore, discovering new natural molecules with high efficiency in controlling plant pathogenic fungal growth is of vital importance to the agricultural sector.

During the Antarctic expedition (ECA59) funded by the Chilean Antarctic Institute, we collected several environmental samples, including soil, lichens, plants, and snow, from the Fildes Peninsula, Antarctica. We isolated 97 fungal strains and examined their diversity and antibiosis ability against two plant pathogens. Through phylogenetic analysis using multi-genetic markers (ITS, LSU, *ACT*, *RPB2*, *TEF1*, and *TUB*) specific to each taxonomic group, we elucidated species diversity with considerable accuracy. Using a dual-culture assay approach, we evaluated the antibiosis potential of all Antarctic fungal strains against *B. cinerea* and *F. culmorum*. Several strains from the genera *Aspergillus*, *Cyathicula*, *Penicillium*, *Pseudeurotium*, *Pseudogymnoascus*, and *Tolypocladium* showed a remarkable capacity to control the growth of these phytopathogens. Thus, our study offers comprehensive insights into the diversity of culturable fungi in Antarctica and their potential for antibiosis. This study will broaden the understanding of Antarctic fungi and establish groundwork for future research.

## Materials and Methods

### Sample collection and processing

**Fig. 1.**
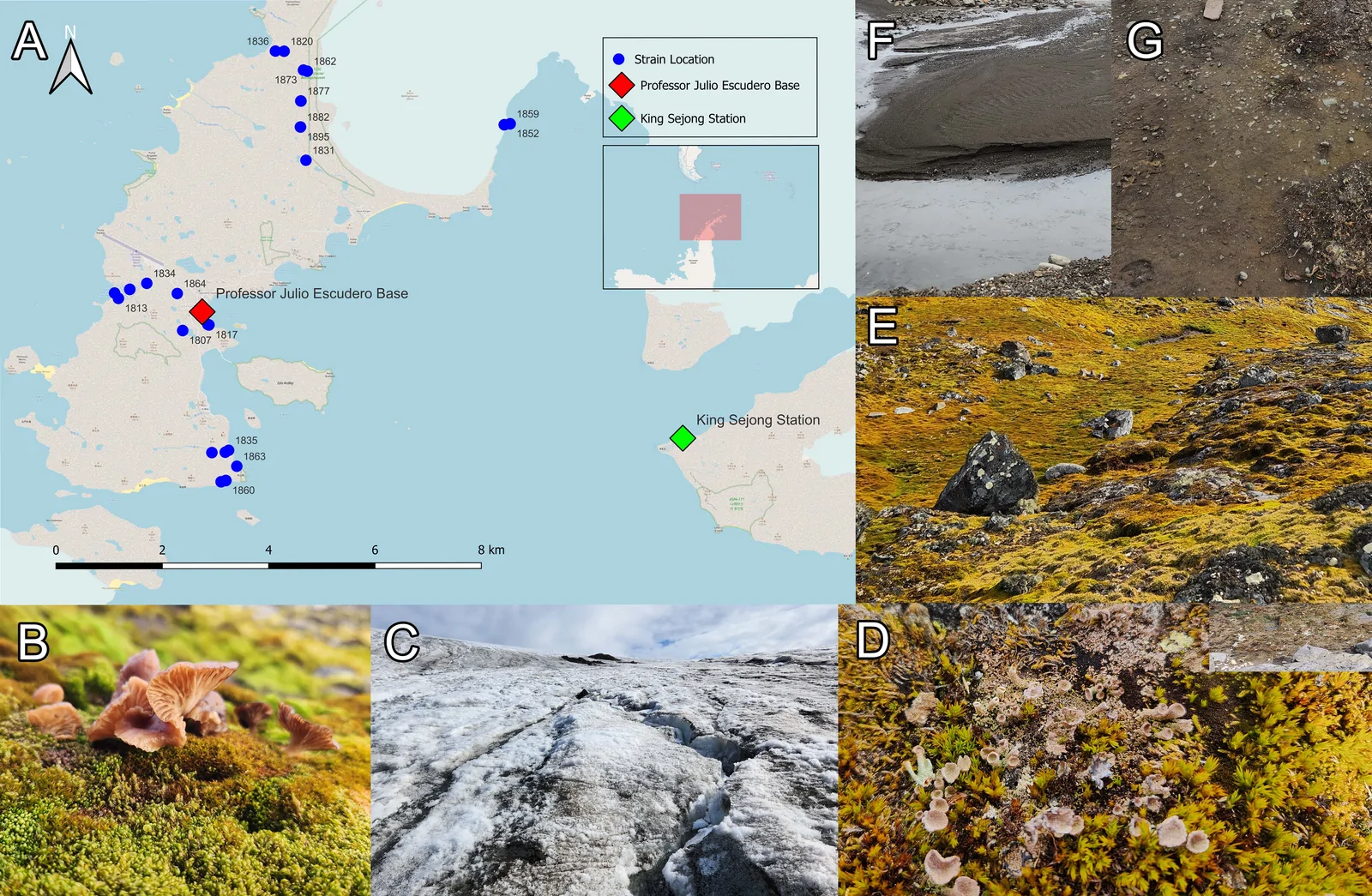
Sampling sites in Antarctica (ECA59 Expedition) with photographs of each sample type. (A) Map indicating the sampling sites with the location of research stations from Chile and South Korea. Representative photographs of (B) fruit body, (C) ice, (D) lichen, (E) moss, (F) sediment, and (G) soil samples.

Antarctic samples for fungal isolation were collected from the Fildes Peninsula, Antarctica in March 2023 (Fig. 1). The exact locations and sample types are listed in Table 1. Samples were collected in sterilized falcon tubes (28×120 mm²) using a metal spatula sterilized with 70% alcohol, transported to Julio Escudero Base Laboratories, and stored at 4°C. They were then transported to the Laboratory of Applied and Sustainable Chemistry (LabQAS; Universidad del Bío-Bío, Chile).

A measured amount (5 g) of each collected sample (sediment, soil, moss, and fruiting body) was resuspended in 10 ml of sterile Type I ultrapure water. From the resulting suspension, 500 µl was plated on Potato Dextrose Agar (PDA; Difco, USA) supplemented with 100 mg/ml tetracycline and 100 mg/ml streptomycin to prevent bacterial contamination and incubated at 13–17°C for one week. Endophytic fungi were isolated from *Deschampsia antarctica* followed the method outlined by Ismail et al. (2021), with some modifications. Briefly, approximately 5 g of root was washed under running tap water to remove any residual soil. Roots that died or showed signs of lesions or discoloration were excluded from the study. The remaining healthy roots were surface sterilized by immersion in 70% ethanol for 3 min, followed by a 2.5 min soak in sodium hypochlorite solution (approximately 5% active chlorine). The roots were then rinsed three times with sterile Type I ultrapure water for 3 min. After surface sterilization, the roots were dried on sterile filter paper and cut into small segments. Seventy root segments per plot were placed on PDA media supplemented with 100 µg/ml tetracycline and 100 mg/ml streptomycin to inhibit bacterial growth. The plates were incubated at 15°C for 4 weeks. The cultures were carefully monitored for fungal mycelia emergence. Once the mycelia were observed, they were immediately transferred to fresh PDA plates to encourage further growth.

Distinct fungal colonies were selected based on their morphological characteristics, including colony color, texture, border type, and radial growth rate. These distinct colonies were then sub-cultured on fresh PDA plates to obtain pure fungal strains. All fungal strains were deposited in the LabQAS Fungal Collection at the Universidad del Bío-Bío, Chile.

### Molecular identification

Genomic DNA was extracted from lyophilized tissues of each fungal strain grown on PDA (using 5 mm diameter blocks) using an AccuPrep Genomic DNA extraction kit (Bioneer Co., Korea), following the manufacturer’s instructions, with a modification of the CTAB buffer instead of the TL buffer. Polymerase chain reaction (PCR) was performed on a C1000 thermal cycler (Bio-Rad, USA) using the AccuPower PCR premix (Bioneer Co., Korea). The primer sets ITS1 and ITS4 (White et al., 1990) were used to amplify the ITS region for all fungal strains under the following conditions: 95°C for 5 min; 35 cycles of 95°C for 40 s, 55°C for 40 s, and 72°C for 1 min; and 72°C for 5 min. All PCR products were verified by gel electrophoresis on a 1% agarose gel and Gel Doc™ XR (Bio-Rad, USA). The PCR products were purified using the Expin™ PCR Purification Kit (GeneAll Biotechnology Co., Korea). DNA sequencing was performed with the same primers used for PCR by Macrogen (Korea), using an ABI PRISM 3700 Genetic Analyzer (Life Technologies, USA). The resulting sequences were proofread and manually edited using Geneious Prime software ver. 2024.0.7 (Biomatters Ltd., USA; Kearse et al., 2012). The forward and reverse sequences obtained were assembled using the de novo assembly function in Geneious Prime software ver. 2024.0.7 (Biomatters Ltd., USA; Kearse et al., 2012).

Preliminary identification at a higher taxonomic level (mostly at the genus level; if not possible, then at the family level) was performed using NCBI BLAST with the ITS region sequences. Based on the preliminary identification *via* NCBI BLAST, appropriate additional genetic markers for each genus were selected through a reference search to allow for species-level identification (Table S1). The PCR conditions for each primer set are summarized in Table S1. The generated sequences were sequenced and edited according to the same protocol used to generate the ITS sequences. All newly generated sequences were deposited in GenBank (Table 1).

For phylogeny-based identification, reference sequences (mostly holotype sequences) were retrieved from GenBank. When holotype sequences were unavailable, verified strain sequences from the published literature were used (Table S2). Using both reference sequences and the newly generated sequences, phylogenetic analyses were performed using FunVIP 0.3.19 with the ‘--preset fast’ setting, employing FastTree for tree construction (https://github.com/Changwanseo/FunVIP; Seo et al., under Review). The set of genetic markers used for the final identification varied depending on the genus. The final species assignment was validated based on phylogenetic evidence, specifically the branch length and local support values of the phylogenetic tree generated using FastTree v.2.1.11 (Price et al., 2010).

**Fig. 2.**
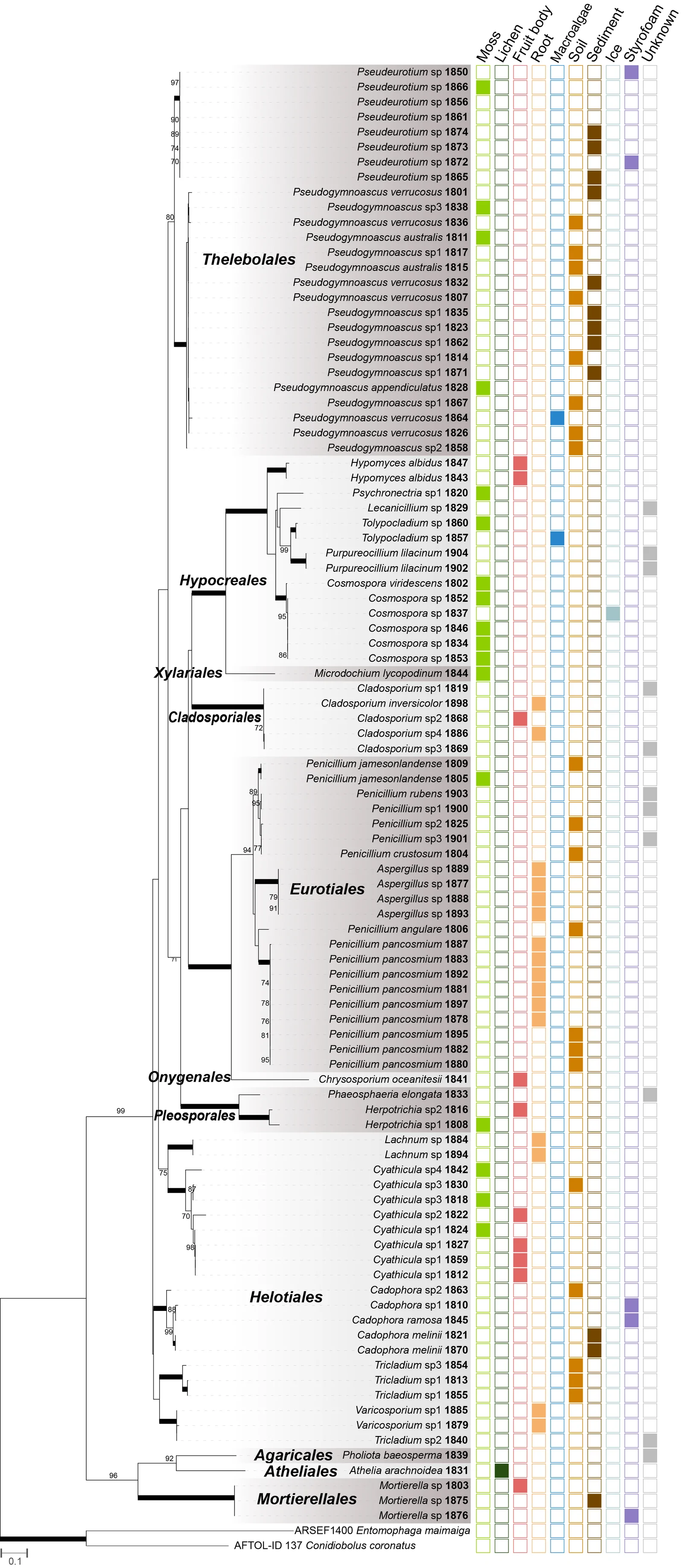
Phylogenetic tree of 97 fungal strains isolated in this study. The phylogenetic tree was constructed using RAxML analysis with internal transcribed spacer (ITS) and LSU sequences. The final identification results for each strain are shown along with the strain numbers in bold. Bootstrap values greater than 70% are indicated at each branch node, and branches with a bootstrap value of 100 are represented by thick lines. The substrate type from which each strain was isolated is indicated next to the strain, with the corresponding type highlighted by a colored box. For clarity, the substrate types are listed at the top of each column.

To construct the phylogenetic tree shown in Fig. 2, RAxML phylogenetic analysis was conducted using the GTR+GAMMA model with 1,000 replicates using RAxML ver. 8 (Stamatakis, 2014). The analysis incorporated the ITS and LSU sequences of the strains obtained in this study, along with two outgroup sequences, *Conidiobolus coronatus* AFTOL-ID 137 and *Entomophaga maimaiga* ARSEF 1400 (Gryganskyi et al., 2012).

### Antibiosis assay employing dual-culture method against *B. cinerea* and *F. culmorum*

Antarctic fungal strains were evaluated against two pathogenic fungi, *B. cinerea* and *F. culmorum*. For in vitro assays, the strain of *B. cinerea* F003 was obtained in 2006 from the blueberry fruit cv. O’Neal, infected with this fungus, in Chillán, Ñuble Region, Chile. The strain was identified based on its microscopic morphological characteristics (presence of conidia and conidiophores) and confirmed by PCR using specific primers Bc3F/R, which amplify the intergenic spacer (IGS) region of the ribosomal DNA of *B. cinerea* (Suarez et al., 2005). The pathogenic isolate of *F. culmorum* strain F066 was isolated from European hazelnut cv. Barcelona in Camarico, Maule Region, Chile. Identification was based on the microscopic morphological characteristics and phylogenetic analysis of the ITS (MT640271), *RPB2* (MT997139), *TEF1* (MT661593), and *CAL* (MT997140) regions (Mishra et al., 2000; O'Donnell et al., 2000, 2008).

Mycelial disks (5 mm in diameter) of Antarctic fungal strains and pathogens were obtained from the margin of an actively growing culture using a cork borer. Both mycelial disks were placed on a Petri dish with 15 ml of PDA and positioned 6 cm apart. The negative controls consisted of mycelial disks from the pathogen alone. The plates were incubated in dark in a culture chamber at 25°C. The percentage of inhibition of radial growth (PIRG) was calculated using the following equation:


$$PIRG\text{\%}=\frac{\left(Dc-Dt\right)}{Dc}\times 100$$

where PIRG is the percentage of growth inhibition,

**Dc** is the growth (mm) of the pathogenic fungus in the control group.

**Dt** is the pathogen growth (mm) in the presence of an Antarctic fungus.

Three replicates were performed for each treatment group. Antagonistic activity was evaluated by measuring the growth radius of the pathogenic fungal mycelia. Once the pathogenic fungus grew free of competition (negative control) and occupied the entire plate, the experiment was terminated. *Fusarium culmorum* and *B. cinerea* occupied the entire plate in 15 and 10 days, respectively.

## Results

### Identification of Antarctic fungi

A total of 97 Antarctic fungal strains were isolated from biotic (moss, lichen, fruit body, macroalgae, and root) and abiotic substrates (soil, sediment, ice, and styrofoam) in similar proportions (Fig. 2), with 48% and 40% of each substrate type, respectively. The substrate type with the highest number of fungal strains was soil (20 strains), followed by moss (18 strains), and roots (16 strains; Table 1).

The ITS region sequences were successfully obtained from 95 of the 97 strains. NCBI BLAST analysis was performed using the ITS region of these 95 strains, whereas the LSU region was used for the remaining two strains. This preliminary analysis identified 97 strains representing 58 taxa. Among these, 54 taxa were assigned to 19 known genera, whereas the remaining four taxa could not be assigned to any known genera. These four taxa matched annotated fungal sequences in the NCBI BLAST database: “*Dothideomycetes* sp.” (strain numbers: 1808, 1816), “*Fungal* sp.” (1818, 1824), “*Helotiales* sp.” (1812, 1813, 1830, 1855, 1859, 1884), and “Uncultured endophytic fungi” (1822, 1842).

Based on previous studies, additional genetic markers suitable for each taxonomic classification were selected, and 132 additional genetic marker sequences were acquired (Table 1): 44 sequences in the LSU region, 34 in the *TUB* region, 7 in the *CMD* region, 10 in the *ACT* region, 34 in the *TEF1* region, and 3 in the *RPB2* region. Phylogenetic analysis using multiple genetic markers was conducted, along with the appropriate reference sequences for each genus. The analysis confirmed that the 58 taxa belonged to three phyla: 6 classes (2 isolates in *Agaricomycetes*, 8 in *Dothideomycetes*, 22 in *Eurotiomycetes*, 47 in *Leotiomycetes*, 3 in *Mortierellomycetes*, and 15 in *Sordariomycetes*), 12 orders (1 isolate in *Agaricales*, 1 in *Amphisphaeriales*, 1 in *Atheliales*, 5 in *Cladosporiales*, 21 in *Eurotiales*, 21 in *Helotiales*, 14 in *Hypocreales*, 3 in *Mortierellales*, 1 in *Onygenales*, 3 in *Pleosporales*, and 26 in *Thelebolales*), 21 families, and 23 genera (Fig. S1). Approximately 30% of the 97 strains (29 strains) were identified at the species-level, whereas the remaining 70% were confirmed as new species candidates, particularly those concentrated in *Leotiomycetes*. The complete strain phylogeny is presented in Fig. 2, based on ITS and LSU sequences, and the final identification results are reflected in the strain annotations.

### Antibiosis evaluation of Antarctic fungi against *B. cinerea* and *F. culmorum*

**Fig. 3.**
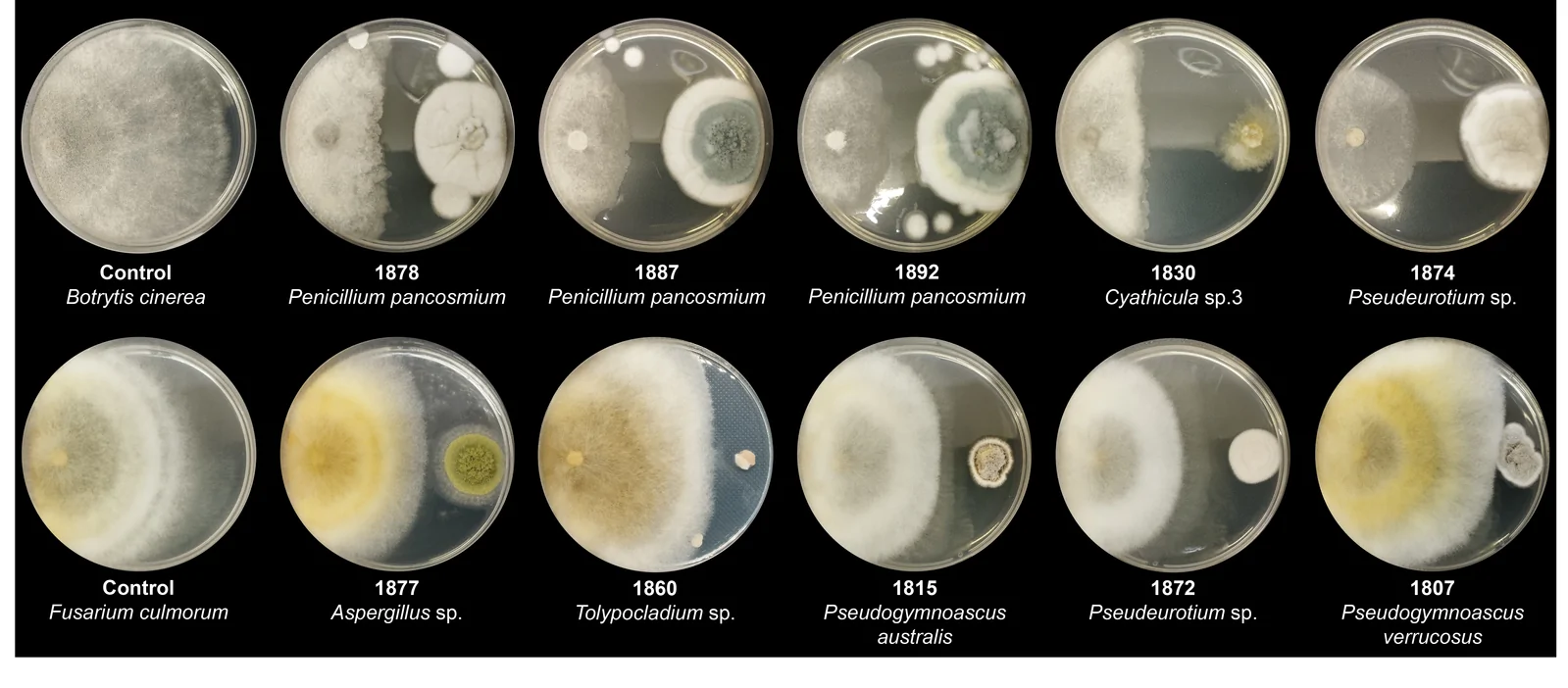
Images showing the top five strains with the highest antibiosis activity against two plant pathogens. The leftmost image in each row represents the control for *B. cinerea* and *F. culmorum*. Strain numbers and identification results are indicated below each plate.

Using a dual-culture assay approach and PIRG as a quantifiable variable, we evaluated the antibiosis potential of all isolated fungal strains against *B. cinerea* and *F. culmorum* (Tables S3 and S4). Overall, remarkable antibiosis bioactivities were observed in the isolated fungal strains, with the best examples shown in Fig. 3.

**Fig. 4.**
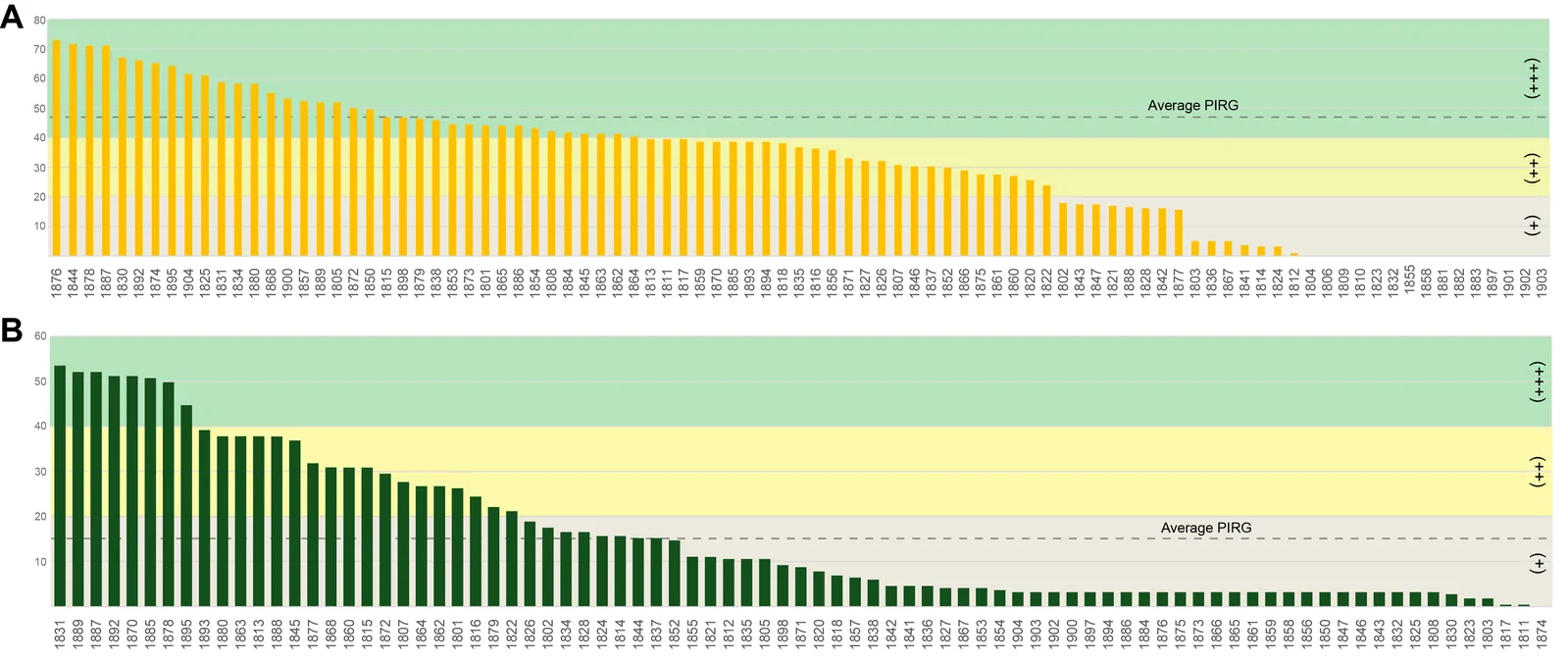
Antibiosis activity of Antarctic fungal strains against two plant pathogens. The graphs show the antibiosis activity results, ranked from highest to lowest, for (A) *B. cinerea* and (B) *F. culmorum*. The average percentage of inhibition of radial growth (PIRG) is indicated by the dashed line in each graph. Antibiosis activity is categorized into four levels, represented by distinct colors: (+++) in green, (++) in yellow, (+) in light gray, and below-average in gray.

The isolated Antarctic fungi exhibited antibiosis activity against *B. cinerea* and *F. culmorum*, with PIRG values ranging from 0 to 72.95% and from 0 to 53.45%, respectively (Tables S3 and S4). Based on the PIRG values, antibiosis activity was categorized into four levels: +++ (PIRG: >40%), ++ (PIRG: 20–40%), + (PIRG: 0–20%), and 0 (no inhibition). The strains showing the highest level of inhibition (+++) included 36 and 8 strains against *B. cinerea* and *F. culmorum*, respectively (Tables S3 and S4). The antibiosis activity of the isolated Antarctic fungi was, on average, higher against *B. cinerea* than against *F. culmorum* (Fig. 4). However, the antibiosis activity of each fungal strain against the two plant pathogenic fungi did not always align consistently.

Three *Penicillium pancosmium* strains, 1878, 1887, and 1892, showed elevated levels of antibiosis against *B. cinerea*, with PIRG values of 71.1, 71.1, and 66, respectively. Additionally, new species candidates of the genera *Cyathicula* (1830) and *Pseudeurotium* (1874) showed remarkable levels of antibiosis activity, with PIRG values of 66.9 and 65.1, respectively (Fig. 4A, Table S3). Against *F. culmorum*, the new species candidates of *Aspergillus* (1877) and *Tolypocladium* (1860) most actively controlled pathogen growth, with PIRG values of 31.8 and 30.8, respectively. In addition, two *Pseudogymnoascus* species (1815 and 1807) and one *Pseudeurotium* strain (1872) controlled *F. culmorum* growth (PIRG = 30.8, 27.6, and 29.5, respectively; Fig. 4B, Table S4).

## Discussion

We successfully isolated 58 diverse fungal taxa at the species level from various regions and substrates in Antarctica, representing the first report of culturable fungi associated with Antarctic fruiting bodies. Additionally, we evaluated the antibiosis potential of all fungal strains isolated during the Antarctic expedition (ECA 59) against *B. cinerea* and *F. culmorum*. This study revealed several Antarctic strains that substantially inhibited the growth of agriculturally relevant fungal pathogens, thereby emphasizing their ecological and biotechnological significance.

A significant number of these isolated fungal strains were identified as new species candidates because they showed no match at the species-level in the existing species databases. This highlights the lack of comprehensive taxonomic studies on Antarctic fungi and their underrepresentation in global databases. Furthermore, discrepancies between the final phylogenetic identification and ITS-based BLAST results were observed, particularly within the orders *Pleosporales* and *Helotiales*. For instance, strains preliminarily identified as “*Fungal* sp.” (1818 and 1824) and “*Helotiales* sp.” (1812, 1830, and 1859), based on ITS-based BLAST, were later classified as *Cyathicula* through phylogenetic analysis. A detailed taxonomic study revealed that the closest known species, *Cyathicula microspora*, shared only 86.1% to 92.3% ITS sequence identity with these Antarctic fungal strains, indicating a substantial genetic divergence. These findings further highlight the limitations of fungal sequence curation in the NCBI database, particularly for the identification of Antarctic fungi, due to the lack of taxonomic studies on these organisms.

Furthermore, the limitations of ITS as the sole marker and the necessity of multi-genetic approaches for accurate fungal taxonomy were also pointed out, when studying for Antarctic fungi. To overcome the taxonomic ambiguities of Antarctic fungi, we applied a multigene marker-based approach to discover new species candidates. This approach is particularly effective for genera such as *Penicillium* and *Cladosporium*, which require additional markers, such as *TUB* and *RPB2*, for reliable species-level classification (Bensch et al., 2012; Visagie et al., 2014). By applying this approach, we resolved taxonomic ambiguities and demonstrated its utility in revealing previously uncharacterized fungal diversity. By providing accurate information on these poorly studied Antarctic fungi, this study contributes to the understanding of their potential impact on the changing Antarctic ecosystem and their hidden capabilities for various future applications.

Taxonomic ambiguities in identifying Antarctic fungi were particularly pronounced in the class *Leotiomycetes*, a group frequently reported in polar environments, including soil, moss, and marine habitats, such as algae, seawater, and sponges (Kochkina et al., 2014, 2019; Ordóñez-Enireb et al., 2022; Rämä et al., 2017; Rosa et al., 2019, 2020). Despite their ecological significance (Bates et al., 2012; Câmara et al., 2021; Kochkina et al., 2014; Park et al., 2015; Yu et al., 2018), *Leotiomycetes* remain understudied, with many unresolved taxonomic issues (Johnston et al., 2019; Quandt and Haelewaters, 2021). This makes the species-level identification particularly difficult for Antarctic *Leotiomycetes* (Henríquez et al., 2014; Hirose et al., 2016, 2017; Kochkina et al., 2014; Ordóñez-Enireb et al., 2022). Recent studies have reported an increasing association between Antarctic mosses and Antarctic *Leotiomycetes* species (De Carvalho et al., 2019; Hirose et al., 2016, 2017), with some *Leotiomycetes* species identified as pathogenic (Rosa et al., 2020, 2021). These findings underscore the need for accurate identification within this class.

Our findings highlight the antifungal potential of Antarctic fungi, many of which are poorly understood. A dual-culture assay revealed significant antifungal activity against two major phytopathogens, *B. cinerea* and *F. culmorum*. On average, *B. cinerea* was more susceptible to the antibiosis effects of the Antarctic fungal isolates than *F. culmorum* (Fig. 4). Fungi belonging to *Eurotiales*, including *Penicillium pancosmium*, exhibit particularly strong antibiosis activity, suggesting their potential as natural fungicides. Although *Penicillium* species are well-documented for their biocontrol activities (Roca-Couso et al., 2021; Thambugala et al., 2020), studies on *P. pancosmium* remain limited, making this a notable discovery.

Among the new species candidates, strains from *Cyathicula* and *Pseudeurotium* showed the highest levels of antibiosis activity against both plant pathogens. To the best of our knowledge, this is the first report of the antifungal activity of *Cyathicula*. Although other species of the family *Helotiaceae*, to which *Cyathicula* belongs, also produce various secondary metabolites with antifungal properties (Chen et al., 2013; Elhamouly et al., 2022), the discovery of such activity in *Cyathicula* expands our understanding of the functional diversity within *Helotiaceae*, highlighting its potential as a source of novel antifungal compounds. Moreover, Antarctic strains of *Aspergillus*, *Penicillium*, *Pseudeurotium*, and *Tolypocladium* exhibited antibiosis activity. These fungal groups were well-known for synthesizing antifungal secondary metabolites (Bladt et al., 2013; Brown et al., 1976; Bushley et al., 2013; Heo et al., 2019; Khokhar et al., 2011; Quandt et al., 2015; Wang et al., 2023).

Notably, *Pseudogymnoascus*, the most taxonomically diverse genus identified in this study (6 taxa, 18 isolates), demonstrated significant antifungal activity, with most showing above-average activity against at least one plant pathogen. This aligns with the results of previous studies indicating the capacity of *Pseudogymnoascus* to synthesize diverse antifungal compounds, such as amphiols, geomycins A–C, and various sesquiterpenes (Antipova et al., 2023; Shi et al., 2021). These findings emphasize their potential as key sources of bioactive compounds and their ecological role in Antarctic environments, where antifungal properties may confer adaptive advantages. This study highlights the immense microbial diversity within Antarctic ecosystems and their potential to be broadly applicable in biotechnology, agriculture, and medicine. The extreme conditions in Antarctica likely drive unique selective pressures, fostering the evolution of microorganisms producing distinctive secondary metabolites (Marx et al., 2007; Núñez-Montero and Barrientos, 2018; Ramasamy et al., 2023).

Therefore, this study improves our understanding of Antarctic fungi by elucidating their diversity across various Antarctic habitats and their antibiosis activity against plant pathogenic fungi. Furthermore, this study highlights the importance of applying multi-genetic approaches for the accurate identification and taxonomic classification of fungi in underexplored regions, such as Antarctica. By identifying new species candidates and characterizing their antibiosis activity against *B. cinerea* and *F. culmorum*, we demonstrate the immense potential of Antarctic fungi as a source of novel bioactive compounds with profound biotechnological applications. As the Antarctic ecosystem continues to undergo changes, this study establishes a foundation for future ecological and biotechnological research by providing critical insights into fungal taxonomy and physiology.

## Figures and Tables

**Table 1.** Collection information and accession numbers of the 97 fungal strains isolated in this study

Order	Family	Species identification	Strain NUM	Substrate	Latitude (S)	Longitude (W)	ITS	LSU	*TUB*	*CMD*	*ACT*	*TEF1*	*RPB2*
Agaricales	Strophariaceae	*Pholiota baeosperma*	1839	-	-	-	PQ427716	PQ427669					
Amphisphaeriales	Amphisphaeriaceae	*Microdochium lycopodinum*	1844	Moss	62°10'11.4168"	58°51'10.2096"	PQ427765	PQ427672					
Atheliales	Atheliaceae	*Athelia arachnoidea*	1831	Lichen	62°10'32.9340"	58°55'28.6860"	PQ427721						
Cladosporiales	Cladosporiaceae	*Cladosporium inversicolor*	1898	Root	62°09'56.8980"	58°55'35.2488"	PQ427783					PQ433547	
		*Cladosporium *sp.1	1819	-	-	-	PQ427699						
		*Cladosporium* sp.2	1868	Fruit body	62°10'10.8408"	58°51'02.6460"	PQ427748				PQ433531	PQ433545	
		*Cladosporium *sp.3	1869	-	-	-	PQ427747						
		*Cladosporium *sp.4	1886	Root	62°09'56.8980"	58°55'35.2488"	PQ427692				PQ433530	PQ433546	
Eurotiales	Aspergillaceae	*Aspergillus *sp.	1877	Root	62°09'56.8980"	58°55'35.2488"	PQ427757		PQ456765				
			1888	Root	62°09'56.8980"	58°55'35.2488"	PQ427709						
			1889	Root	62°09'56.8980"	58°55'35.2488"	PQ427694		PQ456764				
			1893	Root	62°09'56.8980"	58°55'35.2488"	PQ427710						
		*Penicillium angulare*	1806	Soil	62°10'11.4168"	58°51'10.2096"	PQ427704		PQ456772				
		*Penicillium crustosum*	1804	Soil	62°13'48.2520"	58°57'19.5336"	PQ427740		PQ456773				
		*Penicillium jamesonlandense*	1805	Moss	62°10'11.4168"	58°51'10.2096"	PQ427738		PQ456774				
			1809	Soil	62°10'11.4168"	58°51'10.2096"	PQ427739		PQ456775				
		*Penicillium pancosmium*	1878	Root	62°10'11.4168"	58°51'10.2096"	PQ427742		PQ456783				
			1880	Soil	62°10'12.7"	58°55'35.8"	PQ427717		PQ456780	PQ433532			
			1881	Root	62°10'12.7"	58°55'35.8"	PQ427707		PQ456778	PQ433533			
			1882	Soil	62°10'12.7"	58°55'35.8"	PQ427697		PQ456777				
			1883	Root	62°09'56.8980"	58°55'35.2488"	PQ427689		PQ456776	PQ433534			
			1887	Root	62°09'56.8980"	58°55'35.2488"	PQ427737		PQ456782	PQ433535			
			1892	Root	62°09'56.8980"	58°55'35.2488"	PQ427708		PQ456779	PQ433536			
			1895	Soil	62°10'12.7"	58°55'35.8"	PQ427743		PQ456784				
			1897	Root	62°09'56.8980"	58°55'35.2488"	PQ427736		PQ456781	PQ433537			
		*Penicillium rubens*	1903	-	-	-	PQ427741		PQ456785	PQ433538			
		*Penicillium *sp.1	1900	-	-	-	PQ427696		PQ456786				
		*Penicillium *sp.2	1825	Soil	62°12'16.3656"	58°58'09.4368"	PQ427735		PQ456787				
		*Penicillium *sp.3	1901	-	-	-	PQ427773		PQ456788				
Helotiales	Discinellaceae	*Varicosporium* sp.	1879	Root	62°10'11.4168"	58°51'10.2096"	PQ427701	PQ427685					
			1885	Root	62°10'11.4168"	58°51'10.2096"	PQ427763	PQ427686					
	Helotiaceae	*Cyathicula *sp.1	1812	Fruit body	62°13'47.4816"	58°57'13.3560"	PQ427754						
			1824	Moss	62°11'51.3420"	58°59'18.3408"	PQ427753						
			1827	Fruit body	62°13'47.4816"	58°57'13.3560"	PQ427732						
			1859	Fruit body	62°10'10.8408"	58°51'02.6460"	PQ427777						
		*Cyathicula *sp.2	1822	Fruit body	62°12'12.69"	58°57'36.59"	PQ427760						
		*Cyathicula *sp.3	1818	Moss	62°12'12.9708"	58°57'35.2908"	PQ427713						
			1830	Soil	62°12'12.9708"	58°57'35.2908"	PQ427734						
		*Cyathicula *sp.4	1842	Moss	62°09'26.6148"	58°55'57.0360"	PQ427755						
	Lachnaceae	*Lachnum *sp.	1884	Root	62°09'56.8980"	58°55'35.2488"	PQ427781						
			1894	Root	62°10'11.4168"	58°51'10.2096"	PQ427782						
	Ploettnerulaceae	*Cadophora melinii*	1821	Sediment	62°11'51.3420"	58°59'18.3408"	PQ427691	PQ427659	PQ433548				
			1870	Sediment	62°11'51.3420"	58°59'18.3408"	PQ427759		PQ433549				
		*Cadophora ramosa*	1845	Styrofoam	-	-	PQ427705	PQ427673	PQ433550				
		*Cadophora* sp.1	1810	Styrofoam	-	-	PQ427756						
		*Cadophora *sp.2	1863	Soil	62°13'38.8308"	58°56'59.0640"	PQ427690	PQ427679					
	Tricladiaceae	*Tricladium* sp.1	1813	Soil	62°11'56.83"	58°59'33.12"	PQ427714						
		*Tricladium* sp.1	1855	Soil	62°11'56.83"	58°59'33.12"	PQ427719						
		*Tricladium* sp.2	1840	-	-	-		PQ427670					
		*Tricladium* sp.3	1854	Soil	62°12'12.9708"	58°57'35.2908"	PQ427776	PQ427675					
Hypocreales	Cordycipitaceae	*Lecanicillium* sp.	1829	-	-	-	PQ427751	PQ427663					
	Hypocreaceae	*Hypomyces albidus*	1843	Fruit body	62°10'10.8408"	58°51'02.6460"	PQ427770					PQ433543	PQ433539
			1847	Fruit body	62°10'10.8408"	58°51'02.6460"	PQ427766					PQ433544	PQ433540
	Nectriaceae	*Cosmospora viridescens*	1802	Moss	62°13'47.4816"	58°57'13.3560"	PQ427764		PQ456771				
		*Cosmospora* sp.	1834	Moss	62°11'47.6700"	58°58'56.0928"	PQ427768		PQ456770			PQ433542	
			1837	Ice	62°13'30.5256"	58°57'31.5036"	PQ427767		PQ456769				
			1846	Moss	62°13'47.4816"	58°57'13.3560"	PQ427695		PQ456766				
			1852	Moss	62°10'10.8408"	58°51'02.6460"	PQ427726		PQ456768				
			1853	Moss	62°11'51.3420"	58°59'18.3408"	PQ427712		PQ456767				
	Tilachlidiaceae	*Psychronectria *sp.	1820	Moss	62°09'26.6148"	58°55'57.0360"	PQ427700						
	Ophiocordycipitaceae	*Purpureocillium lilacinum*	1902	-	-	-	PQ427702	PQ427687					
			1904	-	-	-	PQ427693	PQ427688					
		*Tolypocladium *sp.	1857	Macroalga	62°10'10.8408"	58°51'02.6460"	PQ427703	PQ427676					
			1860	Moss	62°13'47.4816"	58°57'13.3560"	PQ427728						
Mortierellales	Mortierellaceae	*Mortierella *sp.	1803	Fruit body	62°13'47.4816"	58°57'13.3560"	PQ427746						
			1875	Lagoon sediment	62°12'16.3656"	58°58'09.4368"	PQ427722						
			1876	Styrofoam	-	-	PQ427769	PQ427684					
Onygenales	Onygenaceae	*Chrysosporium *sp.	1841	Fruit body	62°13'47.4816"	58°57'13.3560"	PQ427698	PQ427671					
Pleosporales	Melanommataceae	*Herpotrichia *sp.1	1808	Moss	62°13'47.4816"	58°57'13.3560"	PQ427761	PQ427653				PQ433541	
		*Herpotrichia *sp.2	1816	Fruit body	62°13'47.4816"	58°57'13.3560"	PQ427774	PQ427657					
	Phaeosphaeriaceae	*Phaeosphaeria *sp.	1833	-	-	-		PQ427665					
Thelebolales	Pseudeurotiaceae	*Pseudeurotium *sp.	1850	Styrofoam	-	-	PQ427745	PQ427674					
			1856	Moss	62°12'12.69"	58°57'36.59"	PQ427706						
			1861	Ice	62°13'30.5256"	58°57'31.5036"	PQ427771						
			1865	Sediment	62°12'12.69"	58°57'36.59"	PQ427779						
			1866	Moss	62°15'34.8"	58°59'11.16"	PQ427778	PQ427681					
			1872	Styrofoam	62°12'16.3656"	58°58'09.4368"	PQ427780						
			1873	Sediment	62°09'38.0520"	58°55'31.8540"	PQ427772						
			1874	Sediment	62°15'34.62"	58°59'10.56"	PQ427744						
		*Pseudogymnoascus appendiculatus*	1828	Moss	62°11'51.3420"	58°59'18.3408"	PQ427731	PQ427662					
		*Pseudogymnoascus australis*	1811	Moss	62°13'47.4816"	58°57'13.3560"	PQ427729	PQ427654					
			1815	Soil	62°10'11.4168"	58°51'10.2096"	PQ427727	PQ427656					
		*Pseudogymnoascus verrucosus*	1801	Sediment	62°11'53.5776"	58°59'38.1156"	PQ427752	PQ427651					
			1807	Soil	62°12'16.3656"	58°58'09.4368"	PQ427730	PQ427652					
			1826	Soil	62°12'12.9708"	58°57'35.2908"	PQ427723	PQ427661					
			1832	River sediment	62°13'30.2916"	58°57'14.0508"	PQ427725	PQ427664					
			1836	Soil	62°09'26.4996"	58°56'08.3868"	PQ427758	PQ427667					
			1864	Green alga	62°11'53.8908"	58°58'16.4640"	PQ427750	PQ427680					
		*Pseudogymnoascus* sp.1	1814	Soil	62°13'48.2520"	58°57'19.5336"	PQ427711	PQ427655					
			1817	Soil	62°12'12.9708"	58°57'35.2908"	PQ427720	PQ427658					
			1823	Sediment	62°11'53.5776"	58°59'38.1156"	PQ427724	PQ427660					
			1835	River sediment	62°13'29.1360"	58°57'09.7236"	PQ427733	PQ427666					
			1862	Sediment	62°09'38.7468"	58°55'26.8320"	PQ427749	PQ427678					
			1867	Soil	62°12'12.9708"	58°57'35.2908"	PQ427715	PQ427682					
			1871	River sediment	62°13'30.2916"	58°57'14.0508"	PQ427718	PQ427683					
		*Pseudogymnoascus* sp.2	1858	Soil	62°12'12.9708"	58°57'35.2908"	PQ427762	PQ427677					
		*Pseudogymnoascus* sp.3	1838	Moss	62°11'51.3420"	58°59'18.3408"	PQ427775	PQ427668					

## Supplementary Information

Table S4.

Fig. S1. Phylogenetic trees of the 97 fungal strains classified by each genus. Each tree, from A to W, is organized by genus, with the genus name, type of tree constructed, and the genetic markers used for the phylogenetic analysis indicated. Our strains are highlighted in bold, and the final identification results are marked with boxes.
