# Supplementary material for: Fungal diversity from Fildes Peninsula (Antarctica) and their antibiosis bioactivity against two plant pathogens
Source: J Microbiol. 2025 Apr 14;63(5):e2411029. doi: 10.71150/jm.2411029 (PMC13577079; doi:10.71150/jm.2411029)
Supplement: Supplementary file 1 [file jm-2411029-Supplementary-Fig-S1.pdf]

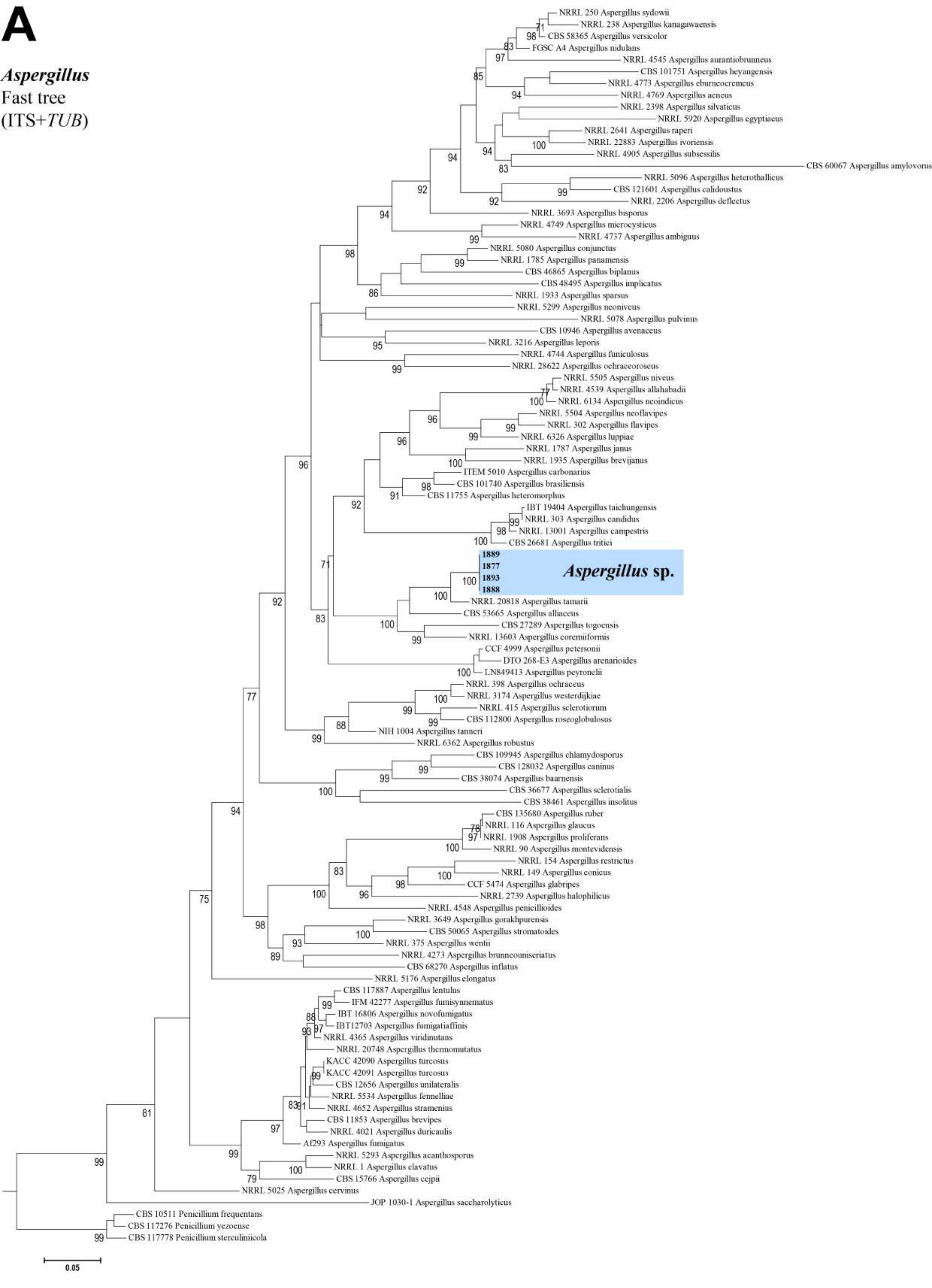
A
Aspergillus
Fast tree
(ITS+TUB)
NRRL 250 Aspergillus sydowii
NRRL 238 Aspergillus kanagawaensis
CBS 58365 Aspergillus versicolor
FGSC A4 Aspergillus nidulans
NRRL 4545 Aspergillus aurantiobrunneus
CBS 101751 Aspergillus heyangensis
NRRL 4773 Aspergillus eburneocremeus
NRRL 4769 Aspergillus aeneus
NRRL 2398 Aspergillus silvaticus
NRRL 5920 Aspergillus egyptiacus
NRRL 2641 Aspergillus raperi
NRRL 22883 Aspergillus ivoriensis
NRRL 4905 Aspergillus subsessilis
CBS 60067 Aspergillus amylovorus
NRRL 5096 Aspergillus heterothallicus
CBS 121601 Aspergillus calidoustus
NRRL 2206 Aspergillus deflectus
NRRL 3693 Aspergillus bisporus
NRRL 4749 Aspergillus microcysticus
NRRL 4737 Aspergillus ambiguus
NRRL 5080 Aspergillus conjunctus
NRRL 1785 Aspergillus panamensis
CBS 46865 Aspergillus biplanus
CBS 48495 Aspergillus implicatus
NRRL 1933 Aspergillus sparsus
NRRL 5299 Aspergillus neoniveus
NRRL 5078 Aspergillus pulvinus
CBS 10946 Aspergillus avenaceus
NRRL 3216 Aspergillus leporis
NRRL 4744 Aspergillus funiculosus
NRRL 28622 Aspergillus ochraceoroseus
NRRL 5505 Aspergillus niveus
NRRL 4539 Aspergillus allahabadii
NRRL 6134 Aspergillus neoindicus
NRRL 5504 Aspergillus neoflavipes
NRRL 302 Aspergillus flavipes
NRRL 6326 Aspergillus luppiae
NRRL 1787 Aspergillus janus
NRRL 1935 Aspergillus brevijanus
ITEM 5010 Aspergillus carbonarius
CBS 101740 Aspergillus brasiliensis
CBS 11755 Aspergillus heteromorphus
IBT 19404 Aspergillus taichungensis
NRRL 303 Aspergillus candidus
NRRL 13001 Aspergillus campestris
CBS 26681 Aspergillus tritici
1889
1877
1893
1888
Aspergillus sp.
NRRL 20818 Aspergillus tamarii
CBS 53665 Aspergillus alliaceus
CBS 27289 Aspergillus togoensis
NRRL 13603 Aspergillus coremiiformis
CCF 4999 Aspergillus petersonii
DTO 268-E3 Aspergillus arenarioides
LN849413 Aspergillus peyronelii
NRRL 398 Aspergillus ochraceus
NRRL 3174 Aspergillus westerdijkiae
NRRL 415 Aspergillus sclerotiorum
CBS 112800 Aspergillus roseoglobulosus
NIH 1004 Aspergillus tanneri
NRRL 6362 Aspergillus robustus
CBS 109945 Aspergillus chlamydosporus
CBS 128032 Aspergillus caninus
CBS 38074 Aspergillus baarnensis
CBS 36677 Aspergillus sclerotialis
CBS 38461 Aspergillus insolitus
CBS 135680 Aspergillus ruber
NRRL 116 Aspergillus glaucus
NRRL 1908 Aspergillus proliferans
NRRL 90 Aspergillus montevidensis
NRRL 154 Aspergillus restrictus
NRRL 149 Aspergillus conicus
CCF 5474 Aspergillus glabripes
NRRL 2739 Aspergillus halophilicus
NRRL 4548 Aspergillus penicillioides
NRRL 3649 Aspergillus gorakhpurensis
CBS 50065 Aspergillus stromatoides
NRRL 375 Aspergillus wentii
NRRL 4273 Aspergillus brunneouniseriatus
CBS 68270 Aspergillus inflatus
NRRL 5176 Aspergillus elongatus
CBS 117887 Aspergillus lentulus
IFM 42277 Aspergillus fumisynnematus
IBT 16806 Aspergillus novofumigatus
IBT12703 Aspergillus fumigatiaffinis
NRRL 4365 Aspergillus viridinutans
NRRL 20748 Aspergillus thermomutatus
KACC 42090 Aspergillus turcosus
KACC 42091 Aspergillus turcosus
CBS 12656 Aspergillus unilateralis
NRRL 5534 Aspergillus fennelliae
NRRL 4652 Aspergillus stramenius
CBS 11853 Aspergillus brevipes
NRRL 4021 Aspergillus duricaulis
Af293 Aspergillus fumigatus
NRRL 5293 Aspergillus acanthosporus
NRRL 1 Aspergillus clavatus
CBS 15766 Aspergillus cejpii
NRRL 5025 Aspergillus cervinus
JOP 1030-1 Aspergillus saccharolyticus
CBS 10511 Penicillium frequentans
CBS 117276 Penicillium yezoense
CBS 117778 Penicillium sterculiniicola
0.05

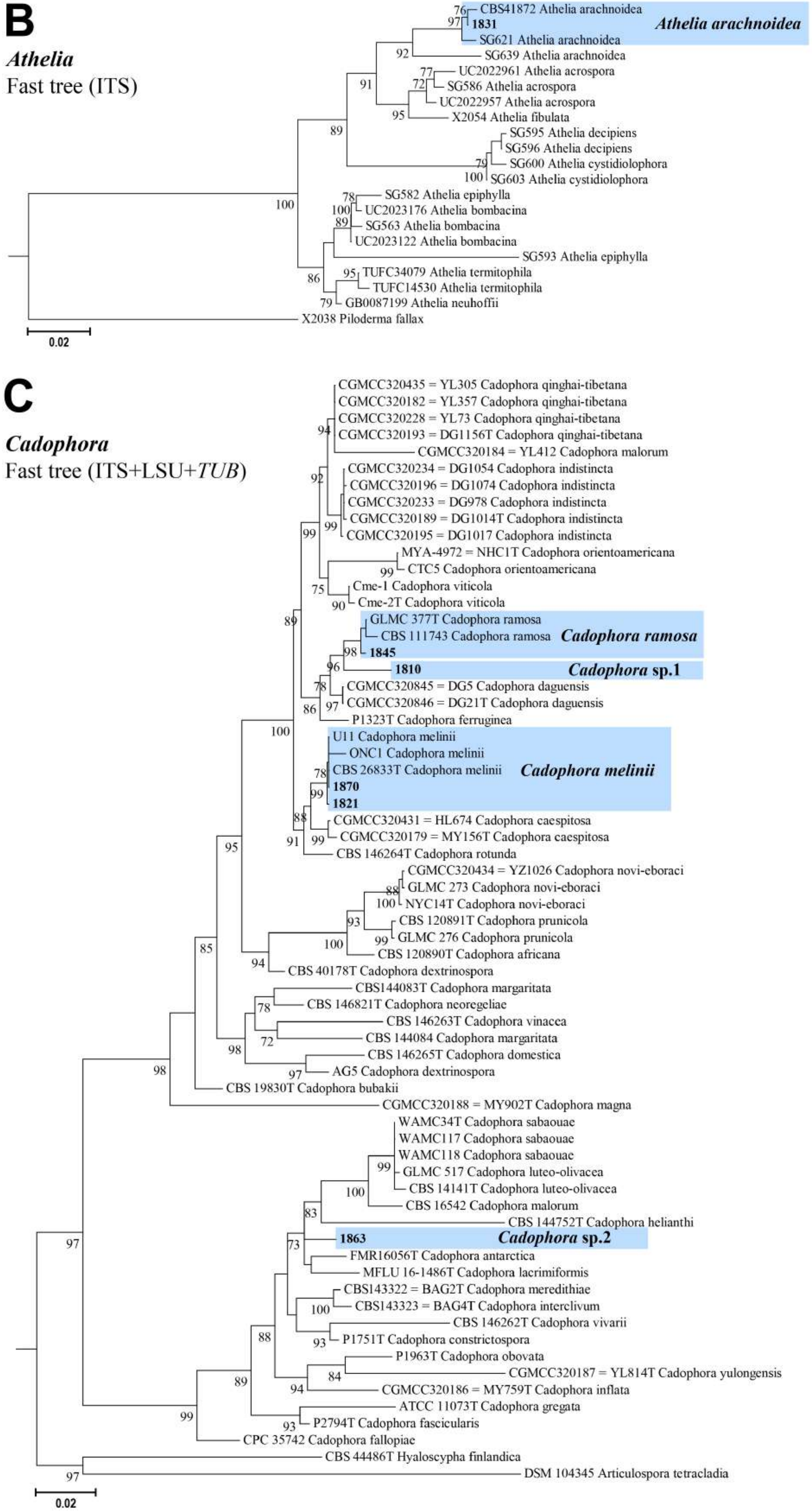

B
Athelia
Fast tree (ITS)
CBS41872 Athelia arachnoidea
1831
SG621 Athelia arachnoidea
Athelia arachnoidea
SG639 Athelia arachnoidea
UC2022961 Athelia acrospora
SG586 Athelia acrospora
UC2022957 Athelia acrospora
X2054 Athelia fibulata
SG595 Athelia decipiens
SG596 Athelia decipiens
SG600 Athelia cystidiolophora
SG603 Athelia cystidiolophora
SG582 Athelia epiphylla
UC2023176 Athelia bombacina
SG563 Athelia bombacina
UC2023122 Athelia bombacina
SG593 Athelia epiphylla
TUFC34079 Athelia termitophila
TUFC14530 Athelia termitophila
GB0087199 Athelia neuhoffii
X2038 Piloderma fallax
76
97
92
91
77
72
95
89
79
100
100
78
100
89
86
95
79
0.02
C
Cadophora
Fast tree (ITS+LSU+TUB)
CGMCC320435 = YL305 Cadophora qinghai-tibetana
CGMCC320182 = YL357 Cadophora qinghai-tibetana
CGMCC320228 = YL73 Cadophora qinghai-tibetana
CGMCC320193 = DG1156T Cadophora qinghai-tibetana
CGMCC320184 = YL412 Cadophora malorum
CGMCC320234 = DG1054 Cadophora indistincta
CGMCC320196 = DG1074 Cadophora indistincta
CGMCC320233 = DG978 Cadophora indistincta
CGMCC320189 = DG1014T Cadophora indistincta
CGMCC320195 = DG1017 Cadophora indistincta
MYA-4972 = NHC1T Cadophora orientoamericana
CTC5 Cadophora orientoamericana
Cme-1 Cadophora viticola
Cme-2T Cadophora viticola
GLMC 377T Cadophora ramosa
CBS 111743 Cadophora ramosa
1845
Cadophora ramosa
1810
Cadophora sp.1
CGMCC320845 = DG5 Cadophora daguensis
CGMCC320846 = DG21T Cadophora daguensis
P1323T Cadophora ferruginea
U11 Cadophora melinii
ONC1 Cadophora melinii
CBS 26833T Cadophora melinii
1870
1821
Cadophora melinii
CGMCC320431 = HL674 Cadophora caespitosa
CGMCC320179 = MY156T Cadophora caespitosa
CBS 146264T Cadophora rotunda
CGMCC320434 = YZ1026 Cadophora novi-eboraci
GLMC 273 Cadophora novi-eboraci
NYC14T Cadophora novi-eboraci
CBS 120891T Cadophora prunicola
GLMC 276 Cadophora prunicola
CBS 120890T Cadophora africana
CBS 40178T Cadophora dextrinospora
CBS144083T Cadophora margaritata
CBS 146821T Cadophora neoregeliae
CBS 146263T Cadophora vinacea
CBS 144084 Cadophora margaritata
CBS 146265T Cadophora domestica
AG5 Cadophora dextrinospora
CBS 19830T Cadophora bubakii
CGMCC320188 = MY902T Cadophora magna
WAMC34T Cadophora sabaouae
WAMC117 Cadophora sabaouae
WAMC118 Cadophora sabaouae
GLMC 517 Cadophora luteo-olivacea
CBS 14141T Cadophora luteo-olivacea
CBS 16542 Cadophora malorum
CBS 144752T Cadophora helianthi
1863
Cadophora sp.2
FMR16056T Cadophora antarctica
MFLU 16-1486T Cadophora lacrimiformis
CBS143322 = BAG2T Cadophora meredithiae
CBS143323 = BAG4T Cadophora interclivum
CBS 146262T Cadophora vivarii
P1751T Cadophora constrictospora
P1963T Cadophora obovata
CGMCC320187 = YL814T Cadophora yulongensis
CGMCC320186 = MY759T Cadophora inflata
ATCC 11073T Cadophora gregata
P2794T Cadophora fascicularis
CPC 35742 Cadophora fallopiae
CBS 44486T Hyaloscypha finlandica
DSM 104345 Articulospora tetracladia
94
92
99
99
99
75
90
89
98
96
78
97
86
100
78
99
88
99
91
95
88
100
93
99
100
85
94
78
72
98
97
98
99
100
83
73
100
93
88
84
94
89
93
99
97
97
0.02

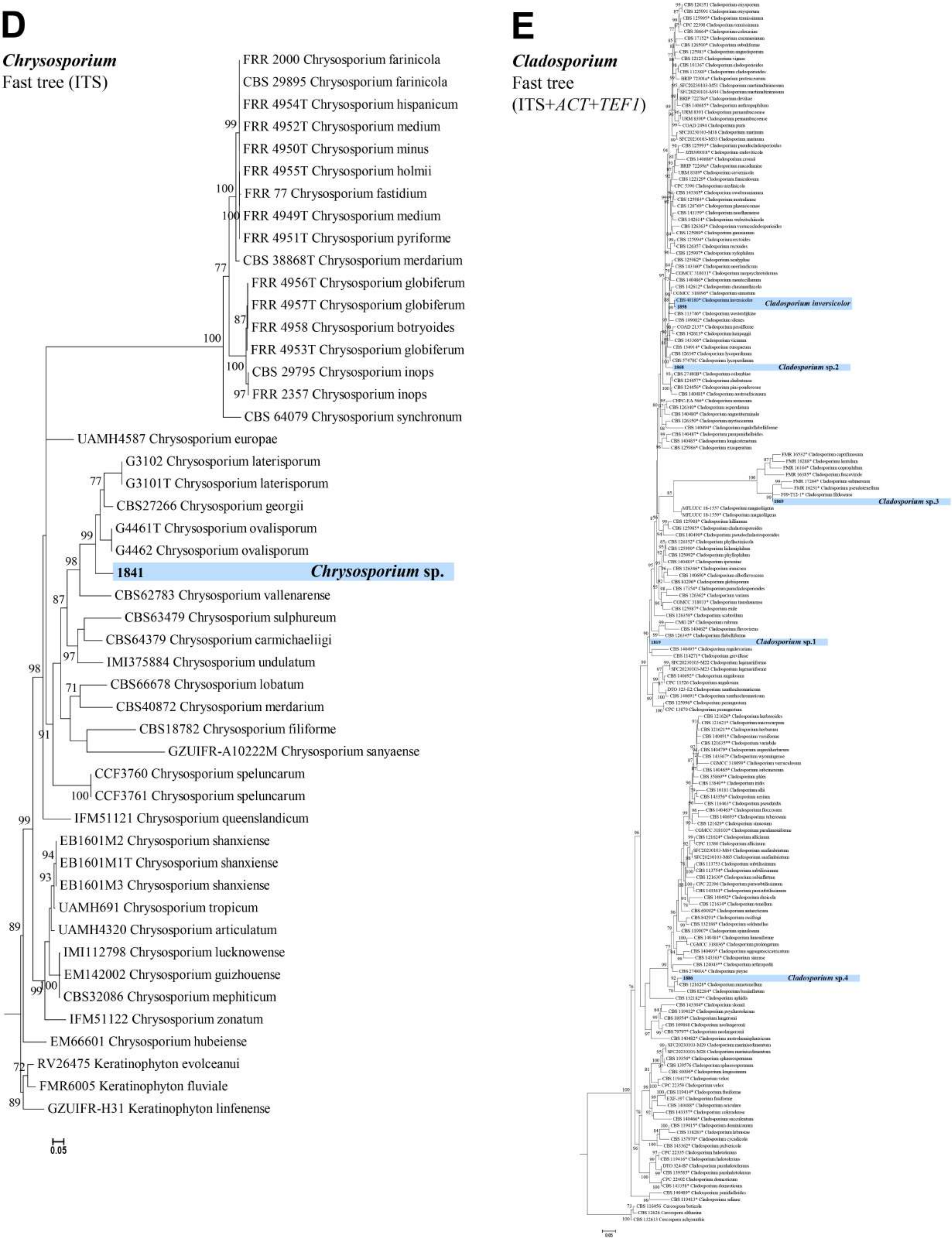

D
Chrysosporium
Fast tree (ITS)
FRR 2000 Chrysosporium farinicola
CBS 29895 Chrysosporium farinicola
FRR 4954T Chrysosporium hispanicum
FRR 4952T Chrysosporium medium
FRR 4950T Chrysosporium minus
FRR 4955T Chrysosporium holmii
FRR 77 Chrysosporium fastidium
FRR 4949T Chrysosporium medium
FRR 4951T Chrysosporium pyriforme
CBS 38868T Chrysosporium merdarium
FRR 4956T Chrysosporium globiferum
FRR 4957T Chrysosporium globiferum
FRR 4958 Chrysosporium botryoides
FRR 4953T Chrysosporium globiferum
CBS 29795 Chrysosporium inops
FRR 2357 Chrysosporium inops
CBS 64079 Chrysosporium synchronum
UAMH4587 Chrysosporium europae
G3102 Chrysosporium laterisporum
G3101T Chrysosporium laterisporum
CBS27266 Chrysosporium georgii
G4461T Chrysosporium ovalisporum
G4462 Chrysosporium ovalisporum
1841 Chrysosporium sp.
CBS62783 Chrysosporium vallenarense
CBS63479 Chrysosporium sulphureum
CBS64379 Chrysosporium carmichaeliigi
IMI375884 Chrysosporium undulatum
CBS66678 Chrysosporium lobatum
CBS40872 Chrysosporium merdarium
CBS18782 Chrysosporium filiforme
GZUIFR-A10222M Chrysosporium sanyaense
CCF3760 Chrysosporium speluncarum
CCF3761 Chrysosporium speluncarum
IFM51121 Chrysosporium queenslandicum
EB1601M2 Chrysosporium shanxiense
EB1601M1T Chrysosporium shanxiense
EB1601M3 Chrysosporium shanxiense
UAMH691 Chrysosporium tropicum
UAMH4320 Chrysosporium articulatum
IMI112798 Chrysosporium lucknowense
EM142002 Chrysosporium guizhouense
CBS32086 Chrysosporium mephiticum
IFM51122 Chrysosporium zonatum
EM66601 Chrysosporium hubeiense
RV26475 Keratinophyton evolceanui
FMR6005 Keratinophyton fluviale
GZUIFR-H31 Keratinophyton linfenense
0.05
E
Cladosporium
Fast tree
(ITS+ACT+TEF1)
Cladosporium inversicolor
Cladosporium sp.2
Cladosporium sp.3
Cladosporium sp.1
Cladosporium sp.4

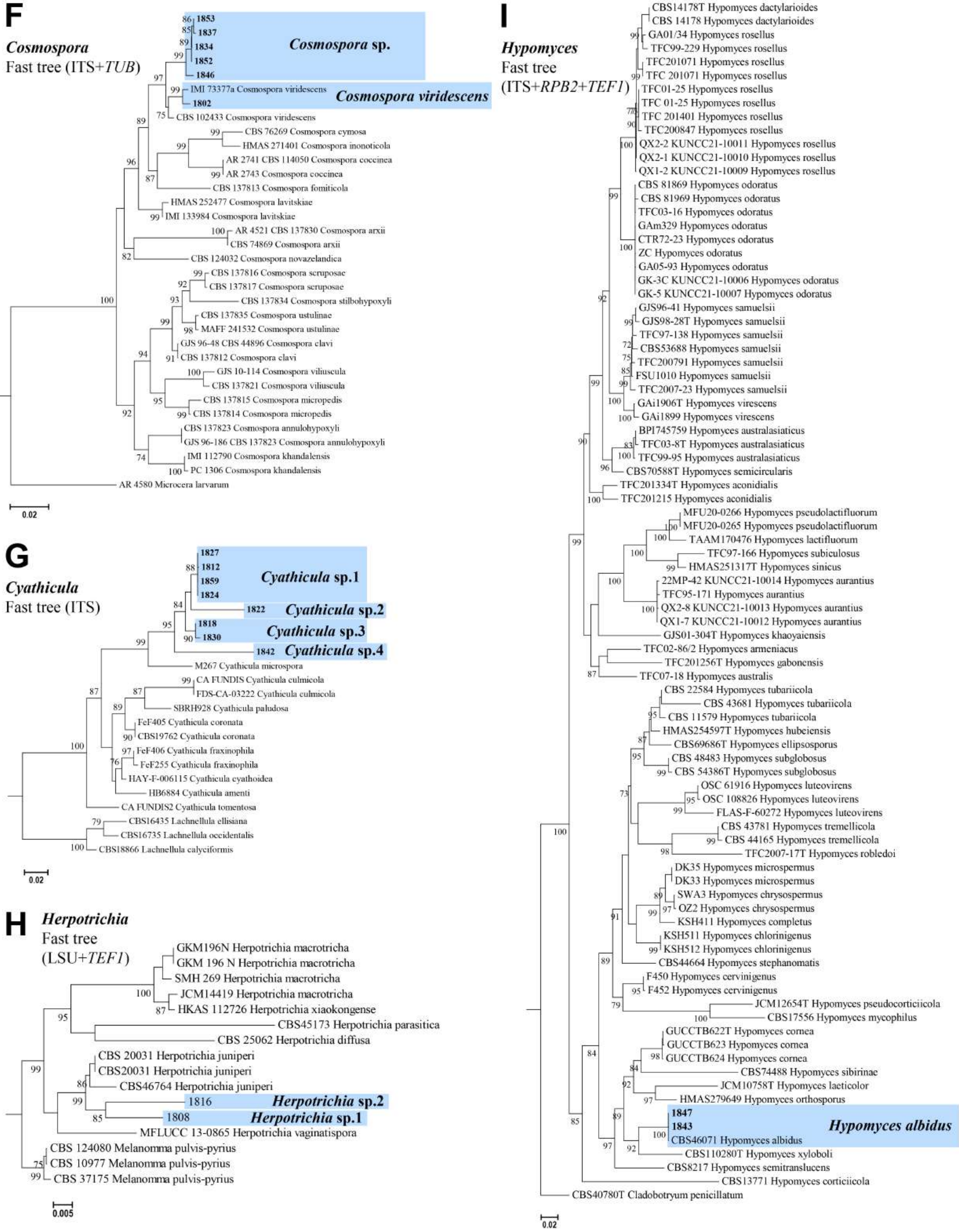

F
Cosmospora
Fast tree (ITS+TUB)
1853
1837
1834
1852
1846
Cosmospora sp.
IMI 73377a Cosmospora viridescens
1802
Cosmospora viridescens
CBS 102433 Cosmospora viridescens
CBS 76269 Cosmospora cymosa
HMAS 271401 Cosmospora inonoticola
AR 2741 CBS 114050 Cosmospora coccinea
AR 2743 Cosmospora coccinea
CBS 137813 Cosmospora fomiticola
HMAS 252477 Cosmospora lavitskiae
IMI 133984 Cosmospora lavitskiae
AR 4521 CBS 137830 Cosmospora arxii
CBS 74869 Cosmospora arxii
CBS 124032 Cosmospora novazelandica
CBS 137816 Cosmospora scruposae
CBS 137817 Cosmospora scruposae
CBS 137834 Cosmospora stilbohypoxyli
CBS 137835 Cosmospora ustulinae
MAFF 241532 Cosmospora ustulinae
GJS 96-48 CBS 44896 Cosmospora clavi
CBS 137812 Cosmospora clavi
GJS 10-114 Cosmospora viliuscula
CBS 137821 Cosmospora viliuscula
CBS 137815 Cosmospora micropedis
CBS 137814 Cosmospora micropedis
CBS 137823 Cosmospora annulohypoxyli
GJS 96-186 CBS 137823 Cosmospora annulohypoxyli
IMI 112790 Cosmospora khandalensis
PC 1306 Cosmospora khandalensis
AR 4580 Microcera larvarum
0.02
G
Cyathicula
Fast tree (ITS)
1827
1812
1859
1824
Cyathicula sp.1
1822
Cyathicula sp.2
1818
1830
Cyathicula sp.3
1842
Cyathicula sp.4
M267 Cyathicula microspora
CA FUNDIS Cyathicula culmicola
FDS-CA-03222 Cyathicula culmicola
SBRH928 Cyathicula paludosa
FeF405 Cyathicula coronata
CBS19762 Cyathicula coronata
FeF406 Cyathicula fraxinophila
FeF255 Cyathicula fraxinophila
HAY-F-006115 Cyathicula cyathoidea
HB6884 Cyathicula amenti
CA FUNDIS2 Cyathicula tomentosa
CBS16435 Lachnellula ellisiana
CBS16735 Lachnellula occidentalis
CBS18866 Lachnellula calyciformis
0.02
H
Herpotrichia
Fast tree
(LSU+TEF1)
GKM196N Herpotrichia macrotricha
GKM 196 N Herpotrichia macrotricha
SMH 269 Herpotrichia macrotricha
JCM14419 Herpotrichia macrotricha
HKAS 112726 Herpotrichia xiaokongense
CBS45173 Herpotrichia parasitica
CBS 25062 Herpotrichia diffusa
CBS 20031 Herpotrichia juniperi
CBS20031 Herpotrichia juniperi
CBS46764 Herpotrichia juniperi
1816
Herpotrichia sp.2
1808
Herpotrichia sp.1
MFLUCC 13-0865 Herpotrichia vaginatispora
CBS 124080 Melanomma pulvis-pyrius
CBS 10977 Melanomma pulvis-pyrius
CBS 37175 Melanomma pulvis-pyrius
0.005
I
Hypomyces
Fast tree
(ITS+RPB2+TEF1)
CBS14178T Hypomyces dactylarioides
CBS 14178 Hypomyces dactylarioides
GA01/34 Hypomyces rosellus
TFC99-229 Hypomyces rosellus
TFC201071 Hypomyces rosellus
TFC 201071 Hypomyces rosellus
TFC01-25 Hypomyces rosellus
TFC 01-25 Hypomyces rosellus
TFC 201401 Hypomyces rosellus
TFC200847 Hypomyces rosellus
QX2-2 KUNCC21-10011 Hypomyces rosellus
QX2-1 KUNCC21-10010 Hypomyces rosellus
QX1-2 KUNCC21-10009 Hypomyces rosellus
CBS 81869 Hypomyces odoratus
CBS 81969 Hypomyces odoratus
TFC03-16 Hypomyces odoratus
GAm329 Hypomyces odoratus
CTR72-23 Hypomyces odoratus
ZC Hypomyces odoratus
GA05-93 Hypomyces odoratus
GK-3C KUNCC21-10006 Hypomyces odoratus
GK-5 KUNCC21-10007 Hypomyces odoratus
GJS96-41 Hypomyces samuelsii
GJS98-28T Hypomyces samuelsii
TFC97-138 Hypomyces samuelsii
CBS53688 Hypomyces samuelsii
TFC200791 Hypomyces samuelsii
FSU1010 Hypomyces samuelsii
TFC2007-23 Hypomyces samuelsii
GAi1906T Hypomyces virescens
GAi1899 Hypomyces virescens
BPI745759 Hypomyces australasiaticus
TFC03-8T Hypomyces australasiaticus
TFC99-95 Hypomyces australasiaticus
CBS70588T Hypomyces semicircularis
TFC201334T Hypomyces aconidialis
TFC201215 Hypomyces aconidialis
MFU20-0266 Hypomyces pseudolactifluorum
MFU20-0265 Hypomyces pseudolactifluorum
TAAM170476 Hypomyces lactifluorum
TFC97-166 Hypomyces subiculosus
HMAS251317T Hypomyces sinicus
22MP-42 KUNCC21-10014 Hypomyces aurantius
TFC95-171 Hypomyces aurantius
QX2-8 KUNCC21-10013 Hypomyces aurantius
QX1-7 KUNCC21-10012 Hypomyces aurantius
GJS01-304T Hypomyces khaoyaiensis
TFC02-86/2 Hypomyces armeniacus
TFC201256T Hypomyces gabonensis
TFC07-18 Hypomyces australis
CBS 22584 Hypomyces tubariicola
CBS 43681 Hypomyces tubariicola
CBS 11579 Hypomyces tubariicola
HMAS254597T Hypomyces hubeiensis
CBS69686T Hypomyces ellipsosporus
CBS 48483 Hypomyces subglobosus
CBS 54386T Hypomyces subglobosus
OSC 61916 Hypomyces luteovirens
OSC 108826 Hypomyces luteovirens
FLAS-F-60272 Hypomyces luteovirens
CBS 43781 Hypomyces tremellicola
CBS 44165 Hypomyces tremellicola
TFC2007-17T Hypomyces robledoi
DK35 Hypomyces microspermus
DK33 Hypomyces microspermus
SWA3 Hypomyces chrysospermus
OZ2 Hypomyces chrysospermus
KSH411 Hypomyces completus
KSH511 Hypomyces chlorinigenus
KSH512 Hypomyces chlorinigenus
CBS44664 Hypomyces stephanomatis
F450 Hypomyces cervinigenus
F452 Hypomyces cervinigenus
JCM12654T Hypomyces pseudocorticiicola
CBS17556 Hypomyces mycophilus
GUCCTB622T Hypomyces cornea
GUCCTB623 Hypomyces cornea
GUCCTB624 Hypomyces cornea
CBS74488 Hypomyces sibirinae
JCM10758T Hypomyces laeticolor
HMAS279649 Hypomyces orthosporus
1847
1843
CBS46071 Hypomyces albidus
Hypomyces albidus
CBS110280T Hypomyces xyloboli
CBS8217 Hypomyces semitranslucens
CBS13771 Hypomyces corticiicola
CBS40780T Cladobotryum penicillatum
0.02

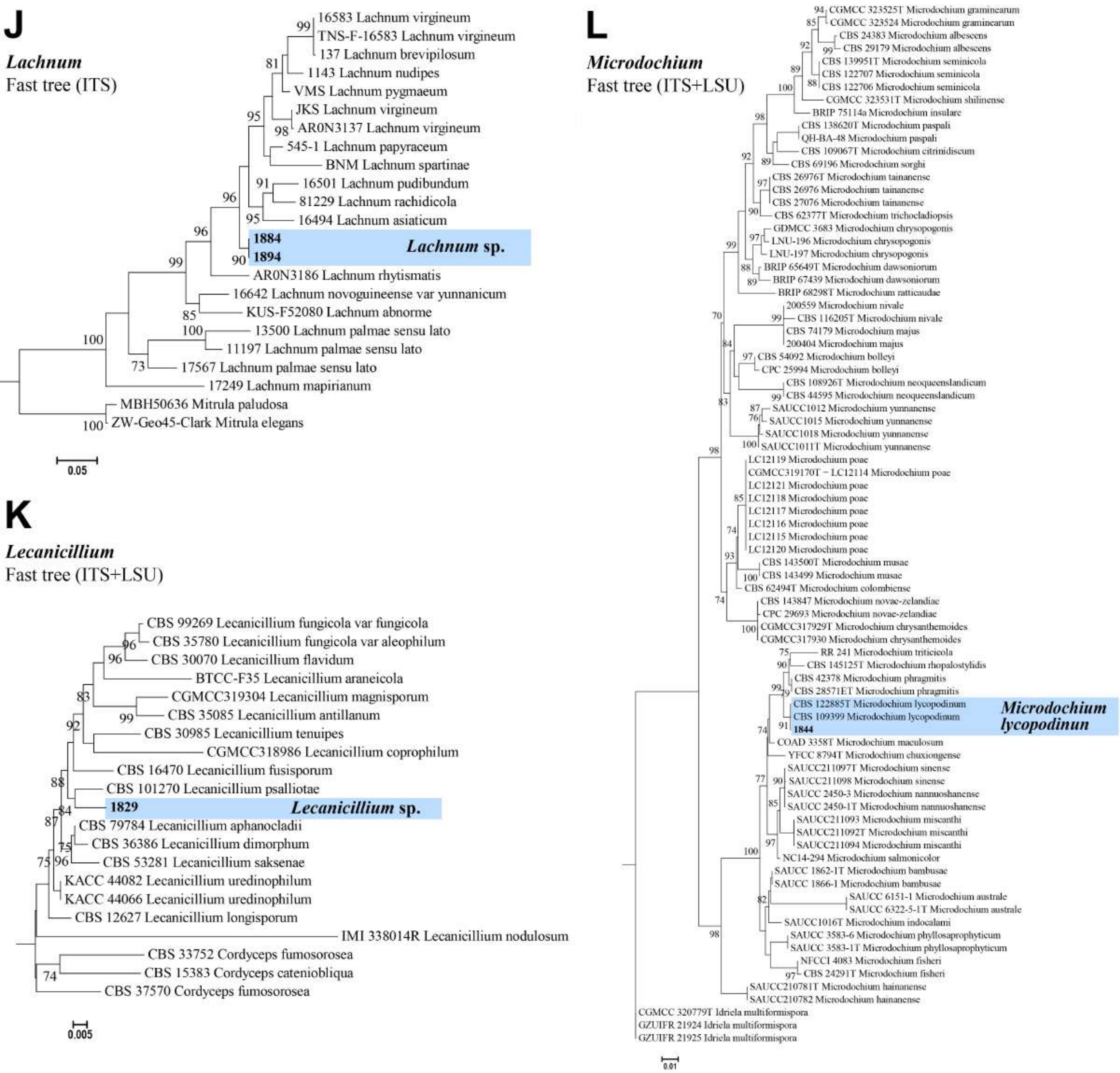

J
Lachnum
Fast tree (ITS)
16583 Lachnum virgineum
TNS-F-16583 Lachnum virgineum
137 Lachnum brevipilosum
1143 Lachnum nudipes
VMS Lachnum pygmaeum
JKS Lachnum virgineum
AR0N3137 Lachnum virgineum
545-1 Lachnum papyraceum
BNM Lachnum spartinae
16501 Lachnum pudibundum
81229 Lachnum rachidicola
16494 Lachnum asiaticum
1884
1894
Lachnum sp.
AR0N3186 Lachnum rhytismatis
16642 Lachnum novoguineense var yunnanicum
KUS-F52080 Lachnum abnorme
13500 Lachnum palmae sensu lato
11197 Lachnum palmae sensu lato
17567 Lachnum palmae sensu lato
17249 Lachnum mapirianum
MBH50636 Mitrula paludosa
ZW-Geo45-Clark Mitrula elegans
0.05
K
Lecanicillium
Fast tree (ITS+LSU)
CBS 99269 Lecanicillium fungicola var fungicola
CBS 35780 Lecanicillium fungicola var aleophilum
CBS 30070 Lecanicillium flavidum
BTCC-F35 Lecanicillium araneicola
CGMCC319304 Lecanicillium magnisporum
CBS 35085 Lecanicillium antillanum
CBS 30985 Lecanicillium tenuipes
CGMCC318986 Lecanicillium coprophilum
CBS 16470 Lecanicillium fusisporum
CBS 101270 Lecanicillium psalliotae
1829
Lecanicillium sp.
CBS 79784 Lecanicillium aphanocladii
CBS 36386 Lecanicillium dimorphum
CBS 53281 Lecanicillium saksenae
KACC 44082 Lecanicillium uredinophilum
KACC 44066 Lecanicillium uredinophilum
CBS 12627 Lecanicillium longisporum
IMI 338014R Lecanicillium nodulosum
CBS 33752 Cordyceps fumosorosea
CBS 15383 Cordyceps cateniobliqua
CBS 37570 Cordyceps fumosorosea
0.005
L
Microdochium
Fast tree (ITS+LSU)
CGMCC 323525T Microdochium graminearum
CGMCC 323524 Microdochium graminearum
CBS 24383 Microdochium albescens
CBS 29179 Microdochium albescens
CBS 139951T Microdochium seminicola
CBS 122707 Microdochium seminicola
CBS 122706 Microdochium seminicola
CGMCC 323531T Microdochium shilinense
BRIP 75114a Microdochium insulare
CBS 138620T Microdochium paspali
QH-BA-48 Microdochium paspali
CBS 109067T Microdochium citrinidiscum
CBS 69196 Microdochium sorghi
CBS 26976T Microdochium tainanense
CBS 26976 Microdochium tainanense
CBS 27076 Microdochium tainanense
CBS 62377T Microdochium trichocladiopsis
GDMCC 3683 Microdochium chrysopogonis
LNU-196 Microdochium chrysopogonis
LNU-197 Microdochium chrysopogonis
BRIP 65649T Microdochium dawsoniorum
BRIP 67439 Microdochium dawsoniorum
BRIP 68298T Microdochium ratticaudae
200559 Microdochium nivale
CBS 116205T Microdochium nivale
CBS 74179 Microdochium majus
200404 Microdochium majus
CBS 54092 Microdochium bolleyi
CPC 25994 Microdochium bolleyi
CBS 108926T Microdochium neoqueenslandicum
CBS 44595 Microdochium neoqueenslandicum
SAUCC1012 Microdochium yunnanense
SAUCC1015 Microdochium yunnanense
SAUCC1018 Microdochium yunnanense
SAUCC1011T Microdochium yunnanense
LC12119 Microdochium poae
CGMCC319170T = LC12114 Microdochium poae
LC12121 Microdochium poae
LC12118 Microdochium poae
LC12117 Microdochium poae
LC12116 Microdochium poae
LC12115 Microdochium poae
LC12120 Microdochium poae
CBS 143500T Microdochium musae
CBS 143499 Microdochium musae
CBS 62494T Microdochium colombiense
CBS 143847 Microdochium novae-zelandiae
CPC 29693 Microdochium novae-zelandiae
CGMCC317929T Microdochium chrysanthemoides
CGMCC317930 Microdochium chrysanthemoides
RR 241 Microdochium triticicola
CBS 145125T Microdochium rhopalostylidis
CBS 42378 Microdochium phragmitis
CBS 28571ET Microdochium phragmitis
CBS 122885T Microdochium lycopodinum
CBS 109399 Microdochium lycopodinum
1844
Microdochium lycopodinun
COAD 3358T Microdochium maculosum
YFCC 8794T Microdochium chuxiongense
SAUCC211097T Microdochium sinense
SAUCC211098 Microdochium sinense
SAUCC 2450-3 Microdochium nannuoshanense
SAUCC 2450-1T Microdochium nannuoshanense
SAUCC211093 Microdochium miscanthi
SAUCC211092T Microdochium miscanthi
SAUCC211094 Microdochium miscanthi
NC14-294 Microdochium salmonicolor
SAUCC 1862-1T Microdochium bambusae
SAUCC 1866-1 Microdochium bambusae
SAUCC 6151-1 Microdochium australe
SAUCC 6322-5-1T Microdochium australe
SAUCC1016T Microdochium indocalami
SAUCC 3583-6 Microdochium phyllosaprophyticum
SAUCC 3583-1T Microdochium phyllosaprophyticum
NFCCI 4083 Microdochium fisheri
CBS 24291T Microdochium fisheri
SAUCC210781T Microdochium hainanense
SAUCC210782 Microdochium hainanense
CGMCC 320779T Idriela multiformispora
GZUIFR 21924 Idriela multiformispora
GZUIFR 21925 Idriela multiformispora
0.01

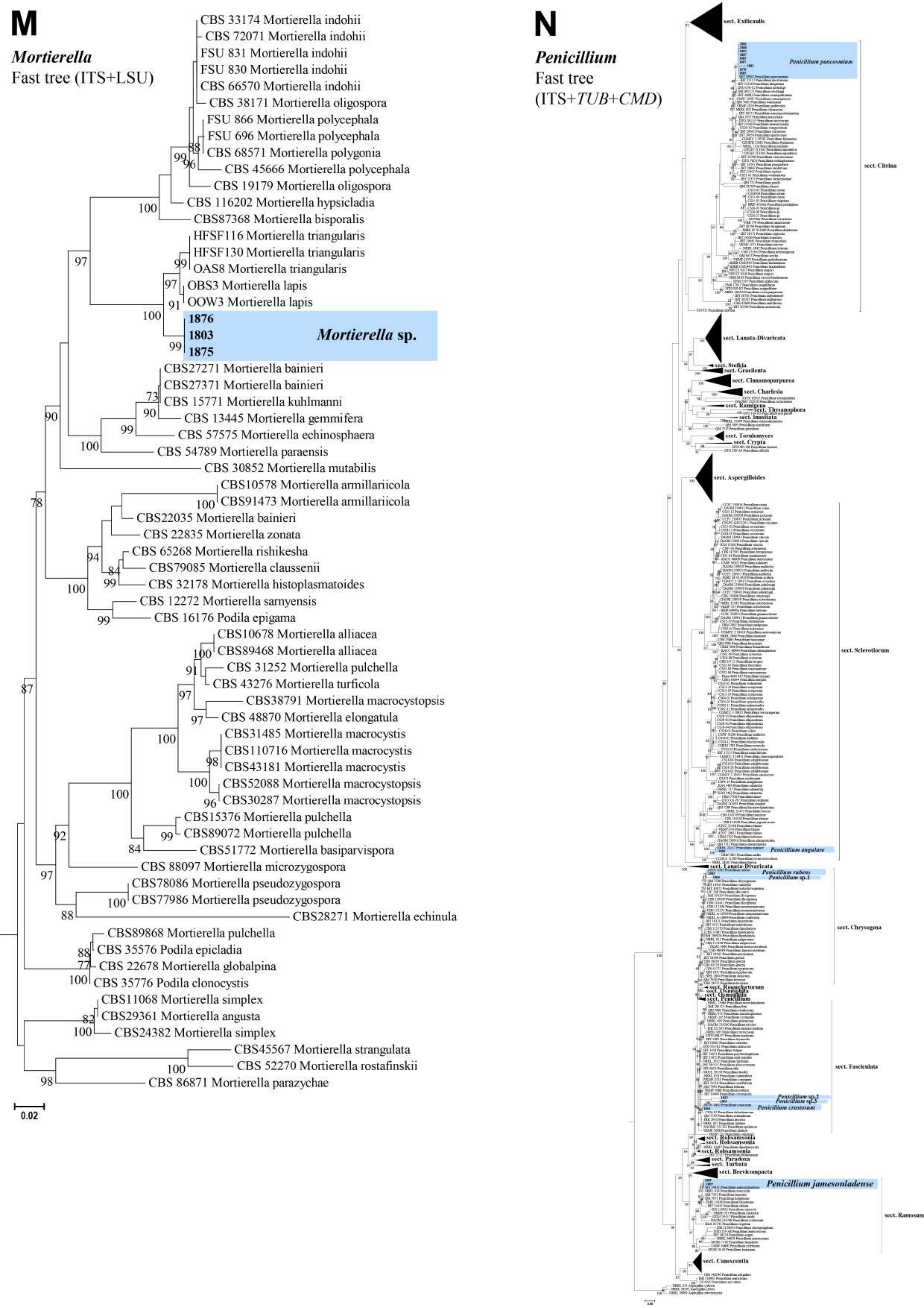
M
Mortierella
Fast tree (ITS+LSU)
CBS 33174 Mortierella indohii
CBS 72071 Mortierella indohii
FSU 831 Mortierella indohii
FSU 830 Mortierella indohii
CBS 66570 Mortierella indohii
CBS 38171 Mortierella oligospora
FSU 866 Mortierella polycephala
FSU 696 Mortierella polycephala
CBS 68571 Mortierella polygonia
CBS 45666 Mortierella polycephala
CBS 19179 Mortierella oligospora
CBS 116202 Mortierella hypsicladia
CBS87368 Mortierella bisporalis
HFSF116 Mortierella triangularis
HFSF130 Mortierella triangularis
OAS8 Mortierella triangularis
OBS3 Mortierella lapis
OOW3 Mortierella lapis
1876
1803
1875
Mortierella sp.
CBS27271 Mortierella bainieri
CBS27371 Mortierella bainieri
CBS 15771 Mortierella kuhlmanni
CBS 13445 Mortierella gemmifera
CBS 57575 Mortierella echinosphaera
CBS 54789 Mortierella paraensis
CBS 30852 Mortierella mutabilis
CBS10578 Mortierella armillariicola
CBS91473 Mortierella armillariicola
CBS22035 Mortierella bainieri
CBS 22835 Mortierella zonata
CBS 65268 Mortierella rishikesha
CBS79085 Mortierella claussenii
CBS 32178 Mortierella histoplasmatoides
CBS 12272 Mortierella sarnyensis
CBS 16176 Podila epigama
CBS10678 Mortierella alliacea
CBS89468 Mortierella alliacea
CBS 31252 Mortierella pulchella
CBS 43276 Mortierella turficola
CBS38791 Mortierella macrocystopsis
CBS 48870 Mortierella elongatula
CBS31485 Mortierella macrocystis
CBS110716 Mortierella macrocystis
CBS43181 Mortierella macrocystis
CBS52088 Mortierella macrocystopsis
CBS30287 Mortierella macrocystopsis
CBS15376 Mortierella pulchella
CBS89072 Mortierella pulchella
CBS51772 Mortierella basiparvispora
CBS 88097 Mortierella microzygospora
CBS78086 Mortierella pseudozygospora
CBS77986 Mortierella pseudozygospora
CBS28271 Mortierella echinula
CBS89868 Mortierella pulchella
CBS 35576 Podila epicladia
CBS 22678 Mortierella globalpina
CBS 35776 Podila clonocystis
CBS11068 Mortierella simplex
CBS29361 Mortierella angusta
CBS24382 Mortierella simplex
CBS45567 Mortierella strangulata
CBS 52270 Mortierella rostafinskii
CBS 86871 Mortierella parazychae
0.02
N
Penicillium
Fast tree
(ITS+TUB+CMD)
sect. Exilicaulis
Penicillium pancosmium
sect. Citrina
sect. Lanata-Divaricata
sect. Stolkia
sect. Gracilenta
sect. Cinnamopurpurea
sect. Charlesia
sect. Ramigena
sect. Thysanophora
sect. Inusitata
sect. Torulomyces
sect. Crypta
sect. Aspergilloides
sect. Sclerotiorum
Penicillium angulare
sect. Lanata-Divaricata
Penicillium rubens
Penicillium sp.1
sect. Chrysogena
sect. Roquefortorum
sect. Osmophila
sect. Penicillium
sect. Fasciculata
Penicillium sp.2
Penicillium sp.3
Penicillium crustosum
sect. Robsamsonia
sect. Robsamsonia
sect. Robsamsonia
sect. Paradoxa
sect. Turbata
sect. Brevicompacta
Penicillium jamesonladense
sect. Ramosum
sect. Canescentia

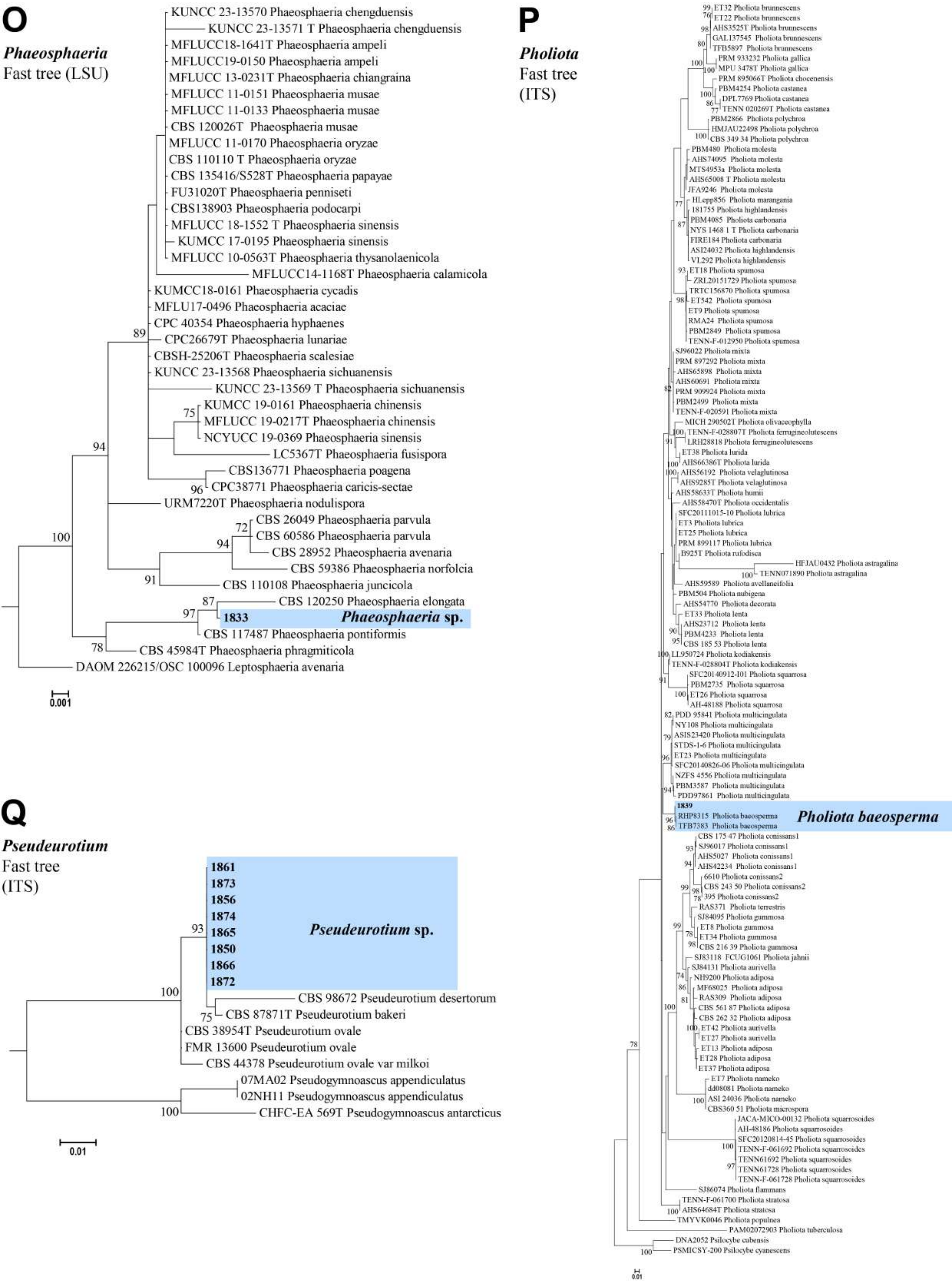
O
Phaeosphaeria
Fast tree (LSU)
KUNCC 23-13570 Phaeosphaeria chengduensis
KUNCC 23-13571 T Phaeosphaeria chengduensis
MFLUCC18-1641T Phaeosphaeria ampeli
MFLUCC19-0150 Phaeosphaeria ampeli
MFLUCC 13-0231T Phaeosphaeria chiangraina
MFLUCC 11-0151 Phaeosphaeria musae
MFLUCC 11-0133 Phaeosphaeria musae
CBS 120026T Phaeosphaeria musae
MFLUCC 11-0170 Phaeosphaeria oryzae
CBS 110110 T Phaeosphaeria oryzae
CBS 135416/S528T Phaeosphaeria papayae
FU31020T Phaeosphaeria penniseti
CBS138903 Phaeosphaeria podocarpi
MFLUCC 18-1552 T Phaeosphaeria sinensis
KUMCC 17-0195 Phaeosphaeria sinensis
MFLUCC 10-0563T Phaeosphaeria thysanolaenicola
MFLUCC14-1168T Phaeosphaeria calamicola
KUMCC18-0161 Phaeosphaeria cycadis
MFLU17-0496 Phaeosphaeria acaciae
CPC 40354 Phaeosphaeria hyphaenes
89
CPC26679T Phaeosphaeria lunariae
CBSH-25206T Phaeosphaeria scalesiae
KUNCC 23-13568 Phaeosphaeria sichuanensis
KUNCC 23-13569 T Phaeosphaeria sichuanensis
75
KUMCC 19-0161 Phaeosphaeria chinensis
MFLUCC 19-0217T Phaeosphaeria chinensis
NCYUCC 19-0369 Phaeosphaeria sinensis
94
LC5367T Phaeosphaeria fusispora
CBS136771 Phaeosphaeria poagena
96
CPC38771 Phaeosphaeria caricis-sectae
URM7220T Phaeosphaeria nodulispora
72
CBS 26049 Phaeosphaeria parvula
CBS 60586 Phaeosphaeria parvula
94
CBS 28952 Phaeosphaeria avenaria
100
CBS 59386 Phaeosphaeria norfolcia
91
CBS 110108 Phaeosphaeria juncicola
87
CBS 120250 Phaeosphaeria elongata
97
1833 Phaeosphaeria sp.
CBS 117487 Phaeosphaeria pontiformis
78
CBS 45984T Phaeosphaeria phragmiticola
DAOM 226215/OSC 100096 Leptosphaeria avenaria
0.001
P
Pholiota
Fast tree (ITS)
ET32 Pholiota brunnescens
ET22 Pholiota brunnescens
AHS3525T Pholiota brunnescens
GAL137545 Pholiota brunnescens
TFB5897 Pholiota brunnescens
PRM 933232 Pholiota gallica
MPU 3478T Pholiota gallica
PRM 895066T Pholiota chocenensis
PBM4254 Pholiota castanea
DPL7769 Pholiota castanea
TENN 020269T Pholiota castanea
PBM2866 Pholiota polychroa
HMJAU22498 Pholiota polychroa
CBS 349 34 Pholiota polychroa
PBM480 Pholiota molesta
AHS74095 Pholiota molesta
MTS4953a Pholiota molesta
AHS65008 T Pholiota molesta
JFA9246 Pholiota molesta
HLepp856 Pholiota marangania
181755 Pholiota highlandensis
PBM4085 Pholiota carbonaria
NYS 1468 1 T Pholiota carbonaria
FIRE184 Pholiota carbonaria
ASI24032 Pholiota highlandensis
VL292 Pholiota highlandensis
ET18 Pholiota spumosa
ZRL20151729 Pholiota spumosa
TRTC156870 Pholiota spumosa
ET542 Pholiota spumosa
ET9 Pholiota spumosa
RMA24 Pholiota spumosa
PBM2849 Pholiota spumosa
TENN-F-012950 Pholiota spumosa
SJ96022 Pholiota mixta
PRM 897292 Pholiota mixta
AHS65898 Pholiota mixta
AHS60691 Pholiota mixta
PRM 909924 Pholiota mixta
PBM2499 Pholiota mixta
TENN-F-020591 Pholiota mixta
MICH 290502T Pholiota olivaceophylla
TENN-F-028807T Pholiota ferrugineolutescens
LRH28818 Pholiota ferrugineolutescens
ET38 Pholiota lurida
AHS66386T Pholiota lurida
AHS56192 Pholiota velaglutinosa
AHS9285T Pholiota velaglutinosa
AHS58633T Pholiota humii
AHS58470T Pholiota occidentalis
SFC20111015-10 Pholiota lubrica
ET3 Pholiota lubrica
ET25 Pholiota lubrica
PRM 899117 Pholiota lubrica
B925T Pholiota rufodisca
HFJAU0432 Pholiota astragalina
TENN071890 Pholiota astragalina
AHS59589 Pholiota avellaneifolia
PBM504 Pholiota nubigena
AHS54770 Pholiota decorata
ET33 Pholiota lenta
AHS23712 Pholiota lenta
PBM4233 Pholiota lenta
CBS 185 53 Pholiota lenta
LL950724 Pholiota kodiakensis
TENN-F-028804T Pholiota kodiakensis
SFC20140912-I01 Pholiota squarrosa
PBM2735 Pholiota squarrosa
ET26 Pholiota squarrosa
AH-48188 Pholiota squarrosa
PDD 95841 Pholiota multicingulata
NY108 Pholiota multicingulata
ASIS23420 Pholiota multicingulata
STDS-1-6 Pholiota multicingulata
ET23 Pholiota multicingulata
SFC20140826-06 Pholiota multicingulata
NZFS 4556 Pholiota multicingulata
PBM3587 Pholiota multicingulata
PDD97861 Pholiota multicingulata
1839
RHP8315 Pholiota baeosperma
TFB7383 Pholiota baeosperma
Pholiota baeosperma
CBS 175 47 Pholiota conissans1
SJ96017 Pholiota conissans1
AHS5027 Pholiota conissans1
AHS42234 Pholiota conissans1
6610 Pholiota conissans2
CBS 243 50 Pholiota conissans2
395 Pholiota conissans2
RAS371 Pholiota terrestris
SJ84095 Pholiota gummosa
ET8 Pholiota gummosa
ET34 Pholiota gummosa
CBS 216 39 Pholiota gummosa
SJ83118 FCUG1061 Pholiota jahnii
SJ84131 Pholiota aurivella
NH9200 Pholiota adiposa
MF68025 Pholiota adiposa
RAS309 Pholiota adiposa
CBS 561 87 Pholiota adiposa
CBS 262 32 Pholiota adiposa
ET42 Pholiota aurivella
ET27 Pholiota aurivella
ET13 Pholiota adiposa
ET28 Pholiota adiposa
ET37 Pholiota adiposa
ET7 Pholiota nameko
dd08081 Pholiota nameko
ASI 24036 Pholiota nameko
CBS360 51 Pholiota microspora
JACA-MICO-00132 Pholiota squarrosoides
AH-48186 Pholiota squarrosoides
SFC20120814-45 Pholiota squarrosoides
TENN-F-061692 Pholiota squarrosoides
TENN61692 Pholiota squarrosoides
TENN61728 Pholiota squarrosoides
TENN-F-061728 Pholiota squarrosoides
SJ86074 Pholiota flammans
TENN-F-061700 Pholiota stratosa
AHS64684T Pholiota stratosa
TMYVK0046 Pholiota populnea
PAM02072903 Pholiota tuberculosa
DNA2052 Psilocybe cubensis
PSMICSY-200 Psilocybe cyanescens
0.01
Q
Pseudeurotium
Fast tree (ITS)
1861
1873
1856
1874
1865
1850
1866
1872
Pseudeurotium sp.
93
100
CBS 98672 Pseudeurotium desertorum
75
CBS 87871T Pseudeurotium bakeri
CBS 38954T Pseudeurotium ovale
FMR 13600 Pseudeurotium ovale
CBS 44378 Pseudeurotium ovale var milkoi
07MA02 Pseudogymnoascus appendiculatus
02NH11 Pseudogymnoascus appendiculatus
100
CHFC-EA 569T Pseudogymnoascus antarcticus
0.01

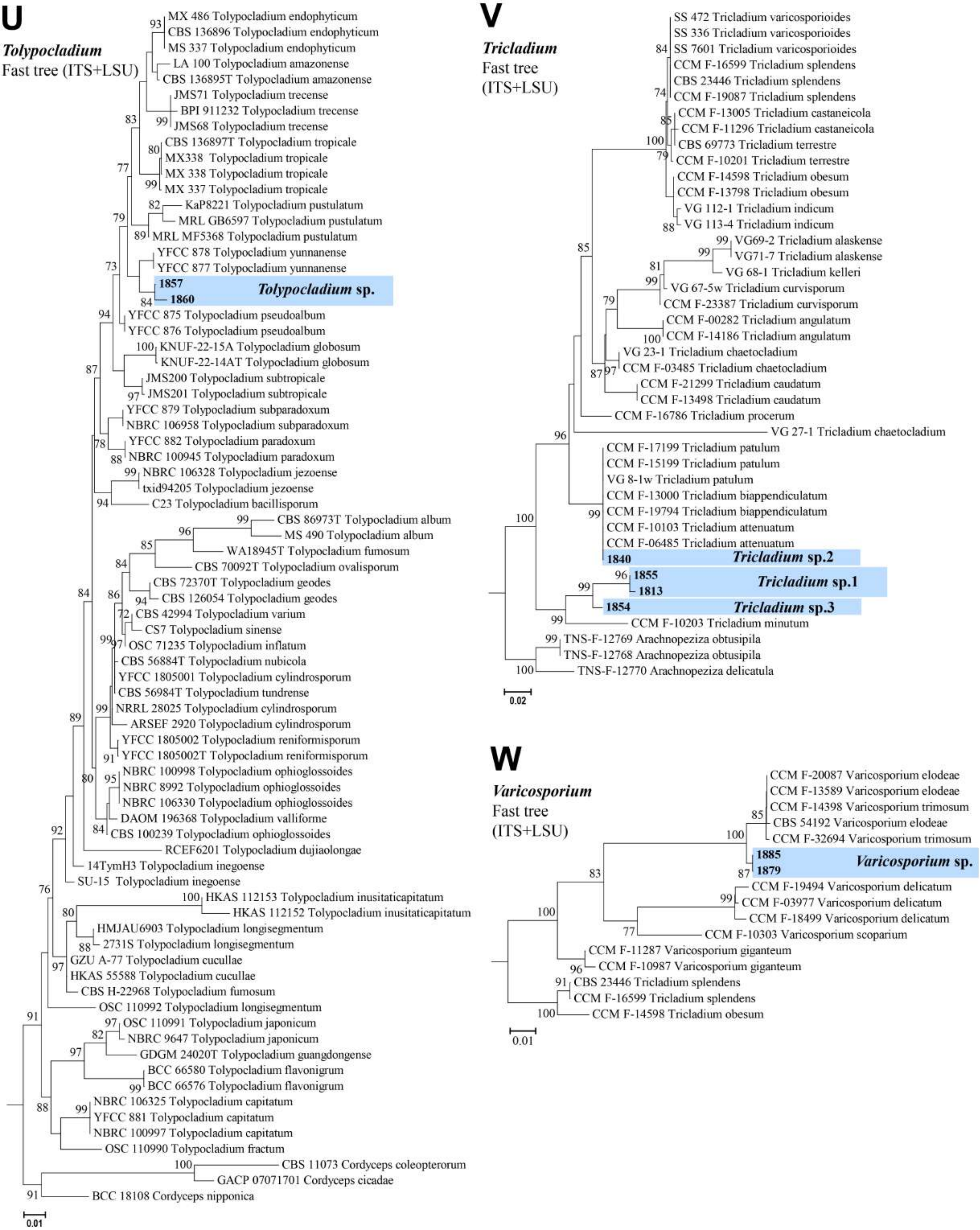
U
Tolypocladium
Fast tree (ITS+LSU)
MX 486 Tolypocladium endophyticum
CBS 136896 Tolypocladium endophyticum
MS 337 Tolypocladium endophyticum
LA 100 Tolypocladium amazonense
CBS 136895T Tolypocladium amazonense
JMS71 Tolypocladium trecense
BPI 911232 Tolypocladium trecense
JMS68 Tolypocladium trecense
CBS 136897T Tolypocladium tropicale
MX338 Tolypocladium tropicale
MX 338 Tolypocladium tropicale
MX 337 Tolypocladium tropicale
KaP8221 Tolypocladium pustulatum
MRL GB6597 Tolypocladium pustulatum
MRL MF5368 Tolypocladium pustulatum
YFCC 878 Tolypocladium yunnanense
YFCC 877 Tolypocladium yunnanense
1857
1860
Tolypocladium sp.
YFCC 875 Tolypocladium pseudoalbum
YFCC 876 Tolypocladium pseudoalbum
KNUF-22-15A Tolypocladium globosum
KNUF-22-14AT Tolypocladium globosum
JMS200 Tolypocladium subtropicale
JMS201 Tolypocladium subtropicale
YFCC 879 Tolypocladium subparadoxum
NBRC 106958 Tolypocladium subparadoxum
YFCC 882 Tolypocladium paradoxum
NBRC 100945 Tolypocladium paradoxum
NBRC 106328 Tolypocladium jezoense
txid94205 Tolypocladium jezoense
C23 Tolypocladium bacillisporum
CBS 86973T Tolypocladium album
MS 490 Tolypocladium album
WA18945T Tolypocladium fumosum
CBS 70092T Tolypocladium ovalisporum
CBS 72370T Tolypocladium geodes
CBS 126054 Tolypocladium geodes
CBS 42994 Tolypocladium varium
CS7 Tolypocladium sinense
OSC 71235 Tolypocladium inflatum
CBS 56884T Tolypocladium nubicola
YFCC 1805001 Tolypocladium cylindrosporum
CBS 56984T Tolypocladium tundrense
NRRL 28025 Tolypocladium cylindrosporum
ARSEF 2920 Tolypocladium cylindrosporum
YFCC 1805002 Tolypocladium reniformisporum
YFCC 1805002T Tolypocladium reniformisporum
NBRC 100998 Tolypocladium ophioglossoides
NBRC 8992 Tolypocladium ophioglossoides
NBRC 106330 Tolypocladium ophioglossoides
DAOM 196368 Tolypocladium valliforme
CBS 100239 Tolypocladium ophioglossoides
RCEF6201 Tolypocladium dujiaolongae
14TymH3 Tolypocladium inegoense
SU-15 Tolypocladium inegoense
HKAS 112153 Tolypocladium inusitaticapitatum
HKAS 112152 Tolypocladium inusitaticapitatum
HMJAU6903 Tolypocladium longisegmentum
2731S Tolypocladium longisegmentum
GZU A-77 Tolypocladium cucullae
HKAS 55588 Tolypocladium cucullae
CBS H-22968 Tolypocladium fumosum
OSC 110992 Tolypocladium longisegmentum
OSC 110991 Tolypocladium japonicum
NBRC 9647 Tolypocladium japonicum
GDGM 24020T Tolypocladium guangdongense
BCC 66580 Tolypocladium flavonigrum
BCC 66576 Tolypocladium flavonigrum
NBRC 106325 Tolypocladium capitatum
YFCC 881 Tolypocladium capitatum
NBRC 100997 Tolypocladium capitatum
OSC 110990 Tolypocladium fractum
CBS 11073 Cordyceps coleopterorum
GACP 07071701 Cordyceps cicadae
BCC 18108 Cordyceps nipponica
0.01
V
Tricladium
Fast tree
(ITS+LSU)
SS 472 Tricladium varicosporioides
SS 336 Tricladium varicosporioides
SS 7601 Tricladium varicosporioides
CCM F-16599 Tricladium splendens
CBS 23446 Tricladium splendens
CCM F-19087 Tricladium splendens
CCM F-13005 Tricladium castaneicola
CCM F-11296 Tricladium castaneicola
CBS 69773 Tricladium terrestre
CCM F-10201 Tricladium terrestre
CCM F-14598 Tricladium obesum
CCM F-13798 Tricladium obesum
VG 112-1 Tricladium indicum
VG 113-4 Tricladium indicum
VG69-2 Tricladium alaskense
VG71-7 Tricladium alaskense
VG 68-1 Tricladium kelleri
VG 67-5w Tricladium curvisporum
CCM F-23387 Tricladium curvisporum
CCM F-00282 Tricladium angulatum
CCM F-14186 Tricladium angulatum
VG 23-1 Tricladium chaetocladium
CCM F-03485 Tricladium chaetocladium
CCM F-21299 Tricladium caudatum
CCM F-13498 Tricladium caudatum
CCM F-16786 Tricladium procerum
VG 27-1 Tricladium chaetocladium
CCM F-17199 Tricladium patulum
CCM F-15199 Tricladium patulum
VG 8-1w Tricladium patulum
CCM F-13000 Tricladium biappendiculatum
CCM F-19794 Tricladium biappendiculatum
CCM F-10103 Tricladium attenuatum
CCM F-06485 Tricladium attenuatum
1840
Tricladium sp.2
1855
1813
Tricladium sp.1
1854
Tricladium sp.3
CCM F-10203 Tricladium minutum
TNS-F-12769 Arachnopeziza obtusipila
TNS-F-12768 Arachnopeziza obtusipila
TNS-F-12770 Arachnopeziza delicatula
0.02
W
Varicosporium
Fast tree
(ITS+LSU)
CCM F-20087 Varicosporium elodeae
CCM F-13589 Varicosporium elodeae
CCM F-14398 Varicosporium trimosum
CBS 54192 Varicosporium elodeae
CCM F-32694 Varicosporium trimosum
1885
1879
Varicosporium sp.
CCM F-19494 Varicosporium delicatum
CCM F-03977 Varicosporium delicatum
CCM F-18499 Varicosporium delicatum
CCM F-10303 Varicosporium scoparium
CCM F-11287 Varicosporium giganteum
CCM F-10987 Varicosporium giganteum
CBS 23446 Tricladium splendens
CCM F-16599 Tricladium splendens
CCM F-14598 Tricladium obesum
0.01
